# Multifactorial Role of Phytogenic CeO_2_ NPs in Crop Improvement and Stress Alleviation: A Mechanistic Overview

**DOI:** 10.1002/pei3.70210

**Published:** 2026-09-15

**Authors:** Faiza Arooj, Khafsa Malik, Tahira Sultana, Abeer Kazmi, Md. Fakhrul Islam, Rafia Azam, Munaza Iram

**Affiliations:** ^1^ Department of Botany Pir Mehr Ali Shah, Arid Agriculture University Rawalpindi Pakistan; ^2^ The State Key Laboratory of Freshwater Ecology and Biotechnology, Institute of Hydrobiology Chinese Academy of Sciences Wuhan Hubei PR China; ^3^ University of Chinese Academy of Sciences Beijing PR China; ^4^ Institute of Emerging Agricultural Technology Shenzhen University of Advanced Technology Shenzhen PR China; ^5^ Jamalpur Science and Technology University Jamalpur Bangladesh

**Keywords:** abiotic stress, biotic stress, CeO_2_ NPs, growth, phytogenic

## Abstract

Numerous biotic and abiotic stresses can affect plants. These stresses negatively affect crop's development, growth, and yield, leading to issues with food security and financial loss. With the development of new technologies like nanotechnology, the negative impacts of environmental stressors on plants have been lessened. After being tested on a variety of agricultural plants, nanotechnology has recently gained a lot of attention since it can improve the health of the soil and crops while minimizing the need for chemical fertilizers. It improves the composition and properties of soil and morphology, physiology, biochemical attributes, and yield of plants. CeO_2_ NPs help plants to grow, manage diseases, promote growth, reduce plant stress, and enhance nutrient management. When CeO_2_ NPs are applied to plants under biotic and abiotic stressors, they trigger stress signaling cascades that scavenge the reactive oxygen species (ROS) (highly reactive oxygen containing species produced as a byproduct of cellular metabolism and can also be produced by environmental stresses) produced and boost plant growth and production. CeO_2_ NPs showed beneficial effects in raising agricultural output by enhancing a variety of physiological and biochemical indicators, which increased yield. This study will provide the latest outlook on the optimum use of CeO_2_ NPs in sustainable agriculture. Moreover, the review article also highlights the impacts of CeO_2_ NPs on biotic and abiotic stress tolerance, plant performance, and the fundamental processes that mitigate environmental stresses. In this review, we discussed the mechanism of uptake, accumulation, stress alleviation, harmful and beneficial effects of CeO_2_ NPs on plants and the environment. A brief overview of the future research that will be focused on more advanced tasks in CeO_2_ NPs, more sustainably.

## Graphical abstract

The graphical abstract illustrates how CeO_2_ nanoparticles help plants withstand biotic and abiotic stresses. Their application reduces excessive ROS through enhanced antioxidant defenses, whereas modulating stress signaling, phytohormones, and gene expression. These responses improve growth, physiological performance, nutrient‐use efficiency, yield, quality, and stress tolerance, supporting sustainable agriculture and food security.

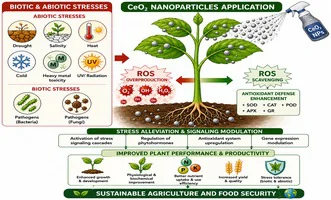

## 1 Introduction

Plants have evolved to survive in their natural habitat, where they are regularly subjected to a wide range of harsh and unpredictable weather conditions. Plants are seriously threatened by biotic stressors like bacteria, fungi, insects, nematodes, and weeds, whereas abiotic stressors include high temperatures, droughts, osmotic pressure, heavy metal contamination, ultraviolet (UV) radiation, and more (Tripathi et al. 2022). Additionally, the quality and productivity of crops may be affected globally by these inevitable plant stresses. These biotic and abiotic stressors consequently reduce agricultural yields and raise the various heavy metals in plant tissues, making them dangerous for humans and animals and leading to serious health consequences (Pandey et al. 2017).

Biotechnology has also been contributing to sustainable agriculture by developing resistant varieties of crops against biotic stresses (insect pests and diseases) and abiotic stresses (drought, cold, flooding, and problem soils). Development of transgenic crops with Plant Growth‐Promoting Rhizobacteria (PGPR) gene expression that are more resilient to a range of biotic and abiotic challenges (Khan et al. 2020). Clustered Regularly Interspaced Short Palindromic Repeats and CRISPR‐associated protein 9 (CRISPR/Cas9), a newly developed genome editing tool, has been extensively associated with perception mutation, gene modification, practical gene analysis, gene knockouts, protein transport on the genome, gene interpretation repression/activation, and epigenome change in a variety of organisms (Ahmed, Iqbal, et al. 2021; Ahmed, Noman, et al. 2021). Nowadays, biotechnology makes it possible to improve plant yields and potential under harsh environmental conditions (Ahmed, Iqbal, et al. 2021; Ahmed, Noman, et al. 2021). However, there are certain negative consequences associated with all of these advanced and modern practices.

These techniques are expensive and require highly skilled technical experts. Considering the current situation and expectations, we require workable, reasonably priced, and socially acceptable solutions to get over these challenges (Sultana et al. 2023). Thus, to lessen the detrimental effects of these stressors on plants, new and cost‐effective strategies must be developed. From recent few decades, nanotechnology have become interest of researchers from different fields (Iqbal et al. 2020). Nanotechnology is multidisciplinary field which have great potential for use in farming that enhance the plants to compete with stresses (Jeelani et al. 2020). Nanoparticles have ability to distribute nutrients and insecticides. Their agricultural use is improving with time. Their availability to crop is increasing because they have vast surface area, small size and strong reactivity, nanoparticles can be easily delivered to specific sites (Jamil et al. 2023). Metal nanoparticles have ability to prevent oxidative stress in plants at lower concentrations and thus enhance plant growth such as silver (Ag), gold (Au) and metal oxides, such as iron oxides (Fe_2_O_3_, Fe_3_O_4_), zinc oxides (ZnO), copper oxide (CuO), titanium dioxide (TiO_2_), and cerium oxide (CeO_2_). Nanoparticles improve the enzymatic activity of many antioxidants, such as superoxide dismutase (SOD) antioxidant enzyme that protects cells from damage caused by oxidative stress, guaiacol peroxidase (GPX) an antioxidant enzyme found primarily in plants that neutralizes dangerous reactive oxygen species (ROS) accumulate during environmental stress, catalase (CAT) an antioxidant enzyme in all organisms that prevents cellular damage and regulates oxidative stress and ascorbate peroxidase (APX) a vital antioxidant enzyme in plants which acts as primary defense system (Venzhik and Deryabin 2023). CeO_2_ NPs have special electrical, optical, and thermal properties (Rajeshkumar and Naik 2018), due to which it have been recently applied in different aspects of agriculture. Cerium is an iron‐gray lustrous element which belongs to the lanthanide group and have an atomic number of 58 and an atomic weight of 140.166 (Khan, Awan, et al. 2022; Khan, Li, et al. 2022; Khan, Mashwani, et al. 2022). Cerium oxide belongs to category that have efficient photocatalytic properties CeO_2_ NPs is cost effective and maintain their catalytic capabilities in challenging conditions (Nyoka et al. 2020). The crystal structure of cerium is face‐centered cubic and have space group of Fm^3^m. It is a semiconductor with a band gap of roughly 3.2 eV (Elahi et al. 2020). Cerium is flexible (hydroscopic) and can oxidize quickly at room temperature, specifically when there is a lot of humidity in environment. Cerium dissolves rapidly in hot water and slowly in cold water (Khan et al. 2020). CeO_2_ NPs have great potential for a wide range of applications in different fields due to their extraordinary physical and chemical properties (Prakash et al. 2021). Cerium oxide is widely used in UV absorbers, oxygen sensors, optical devices, photocatalysts, fuel cells, biocatalysts, and drug delivery (Sabouri et al. 2022). CeO_2_ NPs scavenge reactive oxygen species (ROS) at high concentrations, which are specifically known for their strong oxidizing capabilities in plant and animal models (An et al. 2020). Organization for Economic Cooperation and Development (OECD) has ranked CeO_2_ NPs among the top 13 on its priority list of engineered nanomaterials (ENM).

CeO_2_ NPs can be used as fertilizer and can promote root development, block membrane leakage and peroxidation and trigger antioxidant enzymes (Cao et al. 2017). Low concentrations of cerium can scavenge ROS thus it is beneficial for maintaining cell structure. CeO_2_ can be act as a catalyst in the chlorophyll production and retain the structure of the chloroplast and cell wall (Jurkow et al. 2020). CeO_2_ NPs can enhance plant growth by regulating the defense mechanisms of both enzymatic and non‐enzymatic stressful conditions. There are different methods for the synthesis of CeO_2_ NPs such as physical, chemical, or biological synthesis (Singh et al. 2020). Physical and chemical synthesis of nanoparticles have posed several negative effects on environment due to their toxic metabolites (Gour and Jain 2019). These methods also produce toxic and unstable NPs, due to which their effectiveness diminish (Magudieshwaran et al. 2019). CeO_2_ NPs are synthesized and applied in diverse fields. Green synthesis of CeO_2_ NPs is environment‐friendly approach which involves a biological method, including nutrient‐mediated, polymer‐mediated, algae‐mediated, fungal‐mediated, and plant‐mediated synthesis (Dhall and Self 2018). Microorganisms are a simple, reliable, economical and eco‐friendly method for green synthesis of CeO_2_ nanoparticles (Venkatesh et al. 2016). There are certain disadvantages to use of various living organisms (microorganism species) to prepare cerium oxide NPs as handling of these microbial species can make downstream processing of NPs difficult (Qamar and Ahmad 2021). Certain types of bacteria might have unique needs for development of NPs (Mousa et al. 2021). Nanoparticle preparation could take a long period due microbiological development (Yadav et al. 2020). Hazards could exist due to microbial culture contamination (Carrapiço et al. 2023). To synthesize NPs, the right microorganisms must be chosen, regardless of their enzyme activity and metabolic route. Due to their high energy requirements, high capital costs, and usage of dangerous and poisonous chemicals, these approaches are viewed as unfavorable (Yousof et al. 2022). In this regard, plants are the most effective source because of their quantity, safety, and abundance of stabilizing and reducing chemicals (Singh et al. 2019). Plants are seen to be the greatest potential agents because of their abundance, safety, and non‐harmful nature. In addition to the fact that they are significant producers of secondary metabolites and phytochemicals that pose no chemical risks. Different types of phytochemicals are stored in different plant parts such as leaves, stems, roots, flowers, fruit, pollen, bark, and wood depending on their role in the plant life cycle (Khan, Awan, et al. 2022; Khan, Li, et al. 2022; Khan, Mashwani, et al. 2022).

**FIGURE 1:**
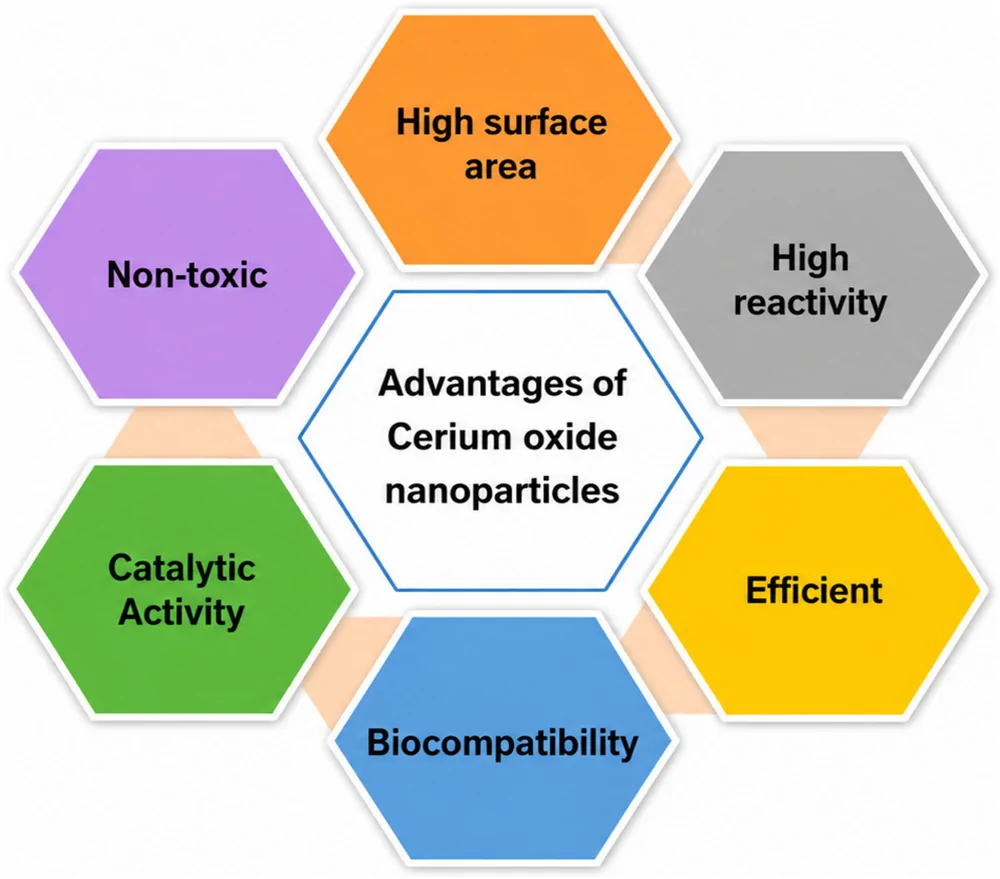
Advantages of phytosynthesized CeO_2_ NPs.

Recently, phytosynthesis of CeO_2_ NPs was reported using different plants such as *Cannabis sativa* , *Equisetum ramosissimum* , *Stevia rebaudiana* , *Ficus religiosa* , and *Jasminum officinale* plant leaf extract (Korkmaz et al. 2024; Mohammed and Hasan 2024; Malakootian et al. 2024). In comparison with other nanoparticles such as Titanium dioxide, gold, copper, and silver, cerium oxide NPs show great potential. Iron, zinc, and titanium NPs are less stable and reactive as compared to CeO_2_ NPs. Iron NPs have a weaker antioxidant property. Iron NPs are prone to oxidation, leading to a decrease in stability. Zinc NPs react with oxygen, causing degradation. Titanium NPs may undergo hydrolysis, affecting stability. The production of highly optimized CeO_2_ NPs using green chemistry is proven to be a successful, economical, and environmentally safe process. CeO_2_ NPs are likely to be regarded as a promising option for increasing fertilizer efficiency in the future. CeO_2_ NPs can be combined with other essential nutrients needed for plant growth to create a biodegradable nanofertilizer (Maqbool 2017). The literature claims that in recent years, the application of biological synthesis of CeO_2_ NPs in various fields has increased in importance because it is inexpensive, quick to synthesize, has a high adsorption capacity, easily recovered, stable, non‐toxic, and harmless to the environment (Figure 1).

This is the only reported overview on the role and applications of CeO_2_ nanoparticles attempted to be explained in this review, which begins with modes of action, biosynthesis strategies, and application in the management of stresses. In conclusion, this comprehensive study has carefully examined synthesis methods, paying particular attention to plant extract‐mediated synthetic methods, the distinct morphological significance, and the diverse range of agricultural applications of biogenic CeO_2_ NPs. Additionally, key details on the mechanisms through which biogenic CeO_2_ NPs reduce biotic and abiotic stresses in crops and the varied application potential of phytosynthesized CeO_2_ NPs in agriculture are also included in this review.

## 2 Fabrication of CeO_2_ NPs

Every day, new methods are used for the synthesis. Research has shown many techniques for oxide‐based nanoparticles formulation for various uses (Nyoka et al. 2020). CeO_2_ NPs can be prepared using a variety of methods, including chemical and biological synthesis (Singh et al. 2020). Every synthesis method adheres to specified guidelines to produce NPs with a given size and form (Dhall and Self 2018). Metal oxide nanoparticles are formulated because of the metal oxide's increased reactivity and efficiency (Ijaz et al. 2020). In nanoparticle synthesis, there are primarily two methods used. The first way, known as the destructive approach, is the top‐down method, which uses large molecules to break down into a tiny form called nanoparticles. The second technique for producing nanoparticles is bottom‐up synthesis, which is sometimes referred to as building‐up synthesis since it uses atoms as the primary building block (Singh et al. 2020) The preparation of nanoparticles can be accomplished by a variety of physical and chemical techniques, including ball milling, condensation and co‐precipitation, hydrothermal, sol–gel, solution combustion, sonochemical, micro emulsion, co‐precipitation, microwave, reverse‐co‐precipitation, and microwave‐hydrothermal (Nosrati et al. 2023; Rajeshkumar and Naik 2018). The synthesis of nanoceria using various nutrients, such as egg white protein, has been documented in several studies (Kargar et al. 2015). CeO_2_ NPs are typically synthesized by using a variety of chemical and/or physical techniques. However, these two approaches may have health risks, and also increase the expense in order to get specific quantity of nanomaterial. Furthermore, it is also observed that the NPs obtained through physical and chemical methods are poisonous and unstable, which makes them less advantageous (Magudieshwaran et al. 2019). The development of “green synthesis” approach solves these problems by making it possible to produce nanoparticles inexpensively using natural resources like plants, microbes, or any kind of organic derivative (Kumar et al. 2018).

Specifically, plant extracts are thought to be an easy and safe method for the synthesis of nanoparticles (Huang et al. 2021). It has been observed that physical and chemical methods for the synthesis of CeO_2_ NPs have a few drawbacks, such as the need for hazardous chemicals, sophisticated automation, and expensive chemicals. The biological approach provides a low‐cost method of nanoparticle synthesis as it utilizes numerous biological species that are found in nature such as plants, algae, fungi, and bacteria. Biological synthesis of nanoparticles has a lot of benefits such as ease of scaling up, non‐toxic, and high yield manufacturing (Vijayaram et al. 2024).

## 3 Phytosynthesis of CeO_2_ NPs

Plants are the most popular, easily available, and inexpensive source for the synthesis of nanoparticles. Compared to fungi, bacteria, and algae, plants provide several benefits in the creation of nanoparticles reported by Pansambal et al. (2023). Every plant has unique phytochemicals that it stores. The phytochemical profiles of different plant species vary. Most plants create these substances to shield themselves from both abiotic and biotic stresses (Singh et al. 2019). Plants contain flavonoids, proteins, carbohydrates, antioxidants, vitamins, and lipids, but some also contain different essential oils and resins (Velgosova et al. 2024). Several factors, including temperature, pH, and the type of plant extract, can influence green synthesis (Anand and Pandey 2023). In earlier studies, scientists generated green synthesized CeO_2_ NPs of varied sizes and forms using a variety of plants (Table 1). In a study, the fruit of *Abelmoschus esculentus* was used for the synthesis of CeO_2_ NPs, which possess a spherical shape and size ranges from 35 to 40 nm. The results show that they have the potential for antibacterial, antioxidant, anticancer, and wound‐healing activities (Ahmed, Iqbal, et al. 2021; Ahmed, Noman, et al. 2021). Different parts of plants are used for the synthesis of CeO_2_ NPs, such as leaves, flowers, roots, and stems. In a study, *Calotropis procera* flower extract is used to synthesize CeO_2_ NPs, which have a spherical shape and have a band gap of 3.29 eV; results show that they have antibacterial properties (Muthuvel, Jothibas, Mohana, and Manoharan 2020; Muthuvel, Jothibas, Manoharan, and Jayakumar 2020). *Prosopis juliflora* leaf extract is utilized to synthesize CeO_2_ NPs having a spherical shape and 15 nm in size, and have the ability to pass through the cell membrane easily (Rajan et al. 2019). A number of researchers have phytosynthesized the CeO_2_ NPs using *S. rebaudiana* (Malakootian et al. 2024), *C. sativa* leaf extracts (Korkmaz et al. 2024), *Ocimum sanctum* (Munirathnam et al. 2023), through the fruit of *Acacia concinna* (Muduli et al. 2023), through the flower of *Hibiscus Rosa sinensis* (Alexander et al. 2023), and *F. religiosa* (Parab et al. 2024) that have active potential for antimicrobial activities.

**TABLE 1:** Antimicrobial and biomedical potential CeO_2_ NPs.

Sr. no.	Name of species	Extract	Activity	Mode of action	References
1	*Cannabis sativa*	Leaves	Antibacterial	– It has a potent bactericidal effect against pathogenic strains – Enzyme inhibition potency	Korkmaz et al. (2024)
2	*Equisetum ramosissimum*	Leaves	Antibacterial	– Possess photocatalytic capabilities – Triggers the production of ROS	Mohammed and Hasan (2024)
3	Mentha	Leaves	Antifungal and antibacterial activity	Induced the oxidation of subcellular organelles and denaturation of DNA and RNA proteins, which stops the cell cycle	Khan et al. (2024)
4	*Stevia rebaudiana*	Leaves	Antibacterial	– Cause changes in the permeability and respiration of bacteria – Produce ROS	Malakootian et al. (2024)
5	*Ficus religiosa*	Leaves	Antibacterial	Destroy cellular components of a bacterial cell, such as DNA, proteins, and lipids	Parab et al. (2024)
6	*Bacopa monnieri*	Leaves	Antioxidant	Inhibits enzymes that produce ROS, reducing oxidative stress	Gopinath et al. (2023)
7	*Nyctanthes arbortristis*	Leaves	Antioxidant	Activity of SOD and APX increase	Anand and Pandey (2023)
8	*Morinda tinctorial*	Leaves	Antibacterial	Electrostatic attraction and provokes the cell wall disruptions	Ahmad et al. (2023)
9	*Acacia concinna*	Fruit	Antibacterial	Penetrate the bacterial cell and inactivate the cellular proteins and enzymes	Muduli et al. (2023)
10	*Ocimum sanctum* Linn	Leaves	Antifungal	Produce oxidative stress that damages the fungal cell	Munirathnam et al. (2023)
11	*Hibiscus Rosa sinensis*	Flower	Antibacterial	Damage respiratory system results in cell division after cell death	Alexander et al. (2023)
12	*Jacaranda mimosifolia*	Leaves	Antibacterial	Altered cell membrane permeability and ROS generation	Vinutha et al. (2023)
13	*Matricaria recutita*	Flowers & leaves	Antibacterial	Typically linked to reactive oxygen species, which are caused by a rise in surface area and oxygen vacancies	Druzian et al. (2023)
14	*Terminalia catappa*	Leaves	Antibacterial	– Interact with the membrane of the bacterial cell, resulting in cell death. When CeO_2_ NPs adhere to the bacterial membrane, they may interact with the mesosome and cause cellular functions to be disrupted	Ray et al. (2023)
15	*Cucurbita pepo*	Peel	Antioxidant	Scavenge ROS by reacting with them and neutralizing their oxidative stress	
16	*Chenopodium quinoa*	Leaves	Antifungal	– Disrupts the membrane permeability by creating an ionic imbalance – Interact with DNA, resulting in structural alterations that prevent the creation of mRNA and proteins	Alotaibi et al. (2023)
17	*Carica papaya*	Leaves	Antibacterial & antifungal	Contacts with the cell wall, attaching to cytosolic proteins, preventing the absorption of nutrients, and production of ROS, which can target physical structures, metabolic processes, and nucleic acid production, and cause microbial cell death	Joshi et al. (2023)
18	*Mentha royleana*	Leaves	Antioxidant	Induce the expression of antioxidant enzymes, which help protect the cell from oxidative stress	
19	*Cassia gluca*	Flower	Antibacterial	The production of reactive oxygen species (ROS) on the membranes of bacterial cells can alter the basic function of vital proteins and, ultimately, their DNA. Proteins may be destroyed, membrane impermeability may be decreased, and cell death may result	Butt et al. (2022)
20	*Acorus calamus*	Rhizome	Antifungal	Strengthen the plant's defenses against the pathogen and reduce reactive oxygen species (ROS) produced during the plant's stress response	Shahbaz et al. (2022)
21	*Curcuma longa*	Stem, Rhizome	Antioxidant	Stimulate the production of antioxidant enzymes, which aid in defending cells against oxidative damage	Kalaycıoğlu et al. (2022)
22	*Artabotrys hexapetalus*	Leaf	Antibacterial	Enter the membranes of bacteria Due to their large interfacial area, thus enhancing their antibacterial efficiency	Parvathy et al. (2022)
23	*Dillenia indica*	Leaves	Antioxidant	SOD, CAT, APX, and GPX are greatly elevated	Das et al. (2022)
24	*Coffea arabica*	Beans	Antibacterial	Raise the buildup of ROS in bacterial cells, which ultimately results in cell death and growth suppression	Zhang et al. (2025)
25	*Dalbergia sissoo*	Leaves	Antioxidant	Directly scavenge ROS by reacting with them and neutralizing their oxidative potential	Alam et al. (2022)
26	*Manilkara zapota*	Fruit peel	Antimicrobial	– Generate a large quantity of ROS and free radicals, which damage physiology and structure and induce fungal death – Strong electrostatic interaction between the bacterial wall surface and Ce destroys the bacteria's outer layer and results in cell death	Ayodhya et al. (2022)
27	*Punica granatum*	Peel	Antioxidant	SOD, CAT, APX, and GPX are greatly elevated	Sheela et al. (2022)
28	*Aquilegia pubiflora*	Leaves	Antibacterial Antifungal Antioxidant	– Negatively charged bacteria and positively charged nanoparticles – Cells interact electrostatically to produce reactive oxygen species (ROS), which kill bacteria, in addition to preventing bacterial development – ROS causes lipids in cell membranes to oxidatively degrade, which denatures the membrane's permeability and lets potassium ions escape, ultimately leading to cell death in antifungal action	Jan et al. (2020)
29	*Abelmoschus esculentus*	Fruits	Antioxidant Antibacterial	– Invasion into the bacterial interior, their DNA, proteins, and lipids are destroyed, which causes the bacteria to die – Mechanical damage by the direct interaction of the CeO_2_ NPs with the bacterial surface causes the denaturing	Ahmed, Iqbal, et al. (2021) and Ahmed, Noman, et al. (2021)
30	*Moringa oleifera*	Seeds	Molluscicidsal	Produce oxidative stress, which resulted in a decline in fertility, a delay in the snail's development, and ultimately its demise	
31	*Acorus calamus*	Rhizome	Antibacterial	Production of ROS and inhibition of EPS formation	Altaf et al. (2021)
32	*M. oleifera*	Leaf	Antimicrobial	– Enter the bacterial cells and cause them to perish – ROS causes lipids in cell membranes to undergo oxidative damage, which denatures the permeability of the membrane and ultimately results in cell death	Putri et al. (2021)
33	*Azadirachta indica*	Leaf	Antibacterial	Produce ROS – Interact with bacterial DNA, causing structural damage and preventing replication	Sathiyapriya et al. (2020)
34	*Calotropis procera*	Flower	Antibacterial	Produce ROS, which can cause fatal damage to microorganisms	Muthuvel, Jothibas, Mohana, and Manoharan (2020) and Muthuvel, Jothibas, Manoharan, and Jayakumar (2020)
35	*Solanum nigrum*	Leaves	Antibacterial	Produces secondary products that cause damage	Muthuvel, Jothibas, Mohana, and Manoharan (2020) and Muthuvel, Jothibas, Manoharan, and Jayakumar (2020)
36	*Simarouba glauca*	Leaf	Bactericidal	Penetrate microbial cell walls and harm DNA, cell protein, which hinders fungal development and leads to fungal demise	Rajan et al. (2020)
37	*Origanum majorana*	Leaves	Antioxidant activity	The defense system includes antioxidant enzymes like SOD and APX, which neutralize oxidative radicals and maintain plants' balance	
38	*Aloe vera*	Leaves	Drought	CeO2 NPs raise the amount of proline in plants when they are stressed by drought, which causes stress tolerance. Reactive oxygen species (ROS) can directly harm proteins, lipid membranes, and DNA	Boora et al. (2024)
39	*Jasminum officinale*	Flower leaves	Chromium stress	Boosted antioxidant enzymes and lowers H_2_O_2_ levels in maize plants under chromium exposure, hence reducing oxidative stress	Latif et al. (2024)

**FIGURE 2:**
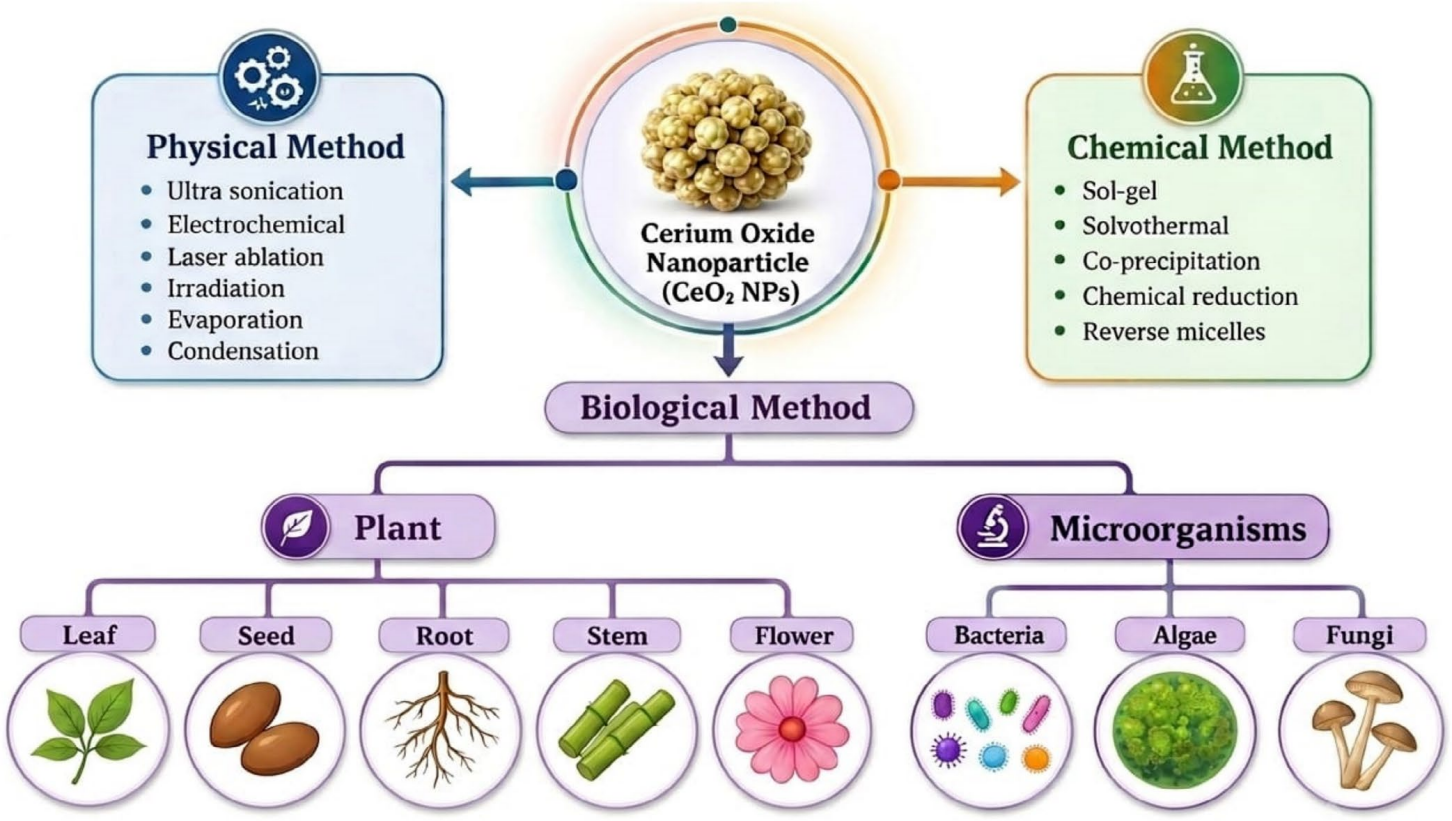
Various methods to synthesize CeO_2_ NPs.

Biological synthesis of CeO_2_ NPs using extracts of different plant parts is a simple process. Ten grams of fresh plant part is added into a 100 mL of distilled water into a 250 mL beaker. The solution is then allowed to heat at 100°C for 10 min. Then filter the extract using Whatman No. 1 filter paper. The aqueous solution of a selected cerium salt was then prepared using double‐distilled water. The produced cerium salt solution and aqueous plant extract are mixed and stirred for 4–8 h at 80°C. After some time, a yellow‐colored precipitate of CeO_2_ NPs formed. The produced CeO_2_ NPs were then separated by centrifugation, cleaned with double‐distilled water, and the precipitate was calcined for 2 h at 500°C. For characterization reasons, yellow‐colored CeO_2_ NPs were therefore meticulously gathered and packaged (Pansambal et al. 2023). Figure 2 demonstrates the different synthesis methods of CeO_2_ NPs.

## 4 Morpho‐Chemical Characterization of CeO_2_ NPs

**FIGURE 3:**
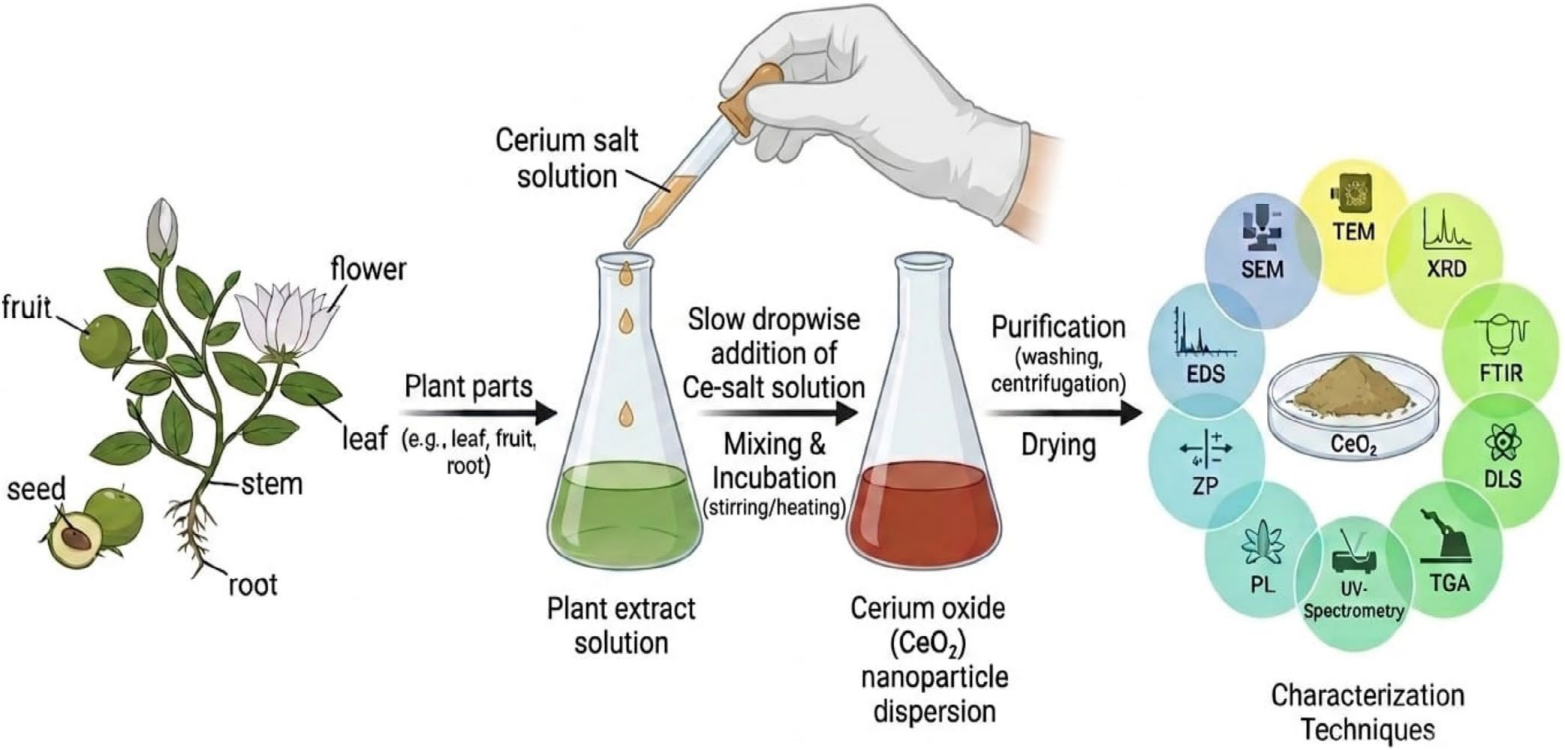
Phytosynthesis and characterization of CeO_2_ NPs.

The biogenic synthesized CeO_2_ NPs were characterized using a variety of methods (Figure 3 demonstrate the phytosynthesis of CeO_2_ NPs and different techniques for characterization), such as energy‐dispersive X‐ray spectroscopy (EDX), scanning electron microscopy (SEM), field emission scanning electron microscopy (FE‐SEM), Fourier transform infrared spectroscopy (FTIR), X‐ray diffraction (XRD), DLS, ZP, and ultraviolet visible spectrometry (UV–Vis) (Oraby 2022) X‐ray photoelectron spectroscopy (XPS), confocal laser scanning microscopy (CLSM), and selected area electron diffraction (SAED) (Venkatesh et al. 2016). Raman spectroscopy, transmission electron microscopy (TEM), and high‐performance liquid chromatography (HPLC) (Jan et al. 2020). CeO_2_ NP production was investigated using UV–vis spectroscopy. CeO_2_ NPs size and crystalline structure were ascertained using the X‐ray diffraction technique. FESEM analysis is used to examine the crystallography and assessment of the NPs gradient chemical composition and morphological picture of CeO_2_ NPs leaf extract mediated CeO_2_ NPs. Transmission electron microscopy is used to analyze morphological characteristics and average size of CeO_2_ NPs. FTIR is used to determine the functional groups of leaf mediated CeO_2_ NPs. Energy‐dispersive X‐ray spectroscopy (EDX) is used to analyze the chemical composition of the substances used to synthesize nanomaterials (Khan, Awan, et al. 2022; Khan, Li, et al. 2022; Khan, Mashwani, et al. 2022) The particle size distribution in the samples was examined using the Dynamic Light Scattering (DLS) approach, which determines the hydrodynamic radius of each particle assuming that they move under Brownian motion like distinct spheres (Mohammed and Hasan 2024). High‐resolution images of the surface morphology and composition of materials is determined by FE‐SEM and optical properties are determined by PL. SAED pattern is used to determine the polycrystalline nature of CeO_2_ NPs. The surface oxidation states of CeO_2_nanoparticles, Ce (3d) and O (1s) were determined by X‐ray Photoelectron Spectroscopy (XPS) research. The effective capping of bioactive substances on the nanoparticles was validated by HPLC experiments.

## 5 Recent Developments in Sustainable Agriculture and Reducing Global Issues Using CeO_2_ NPs

**FIGURE 4:**
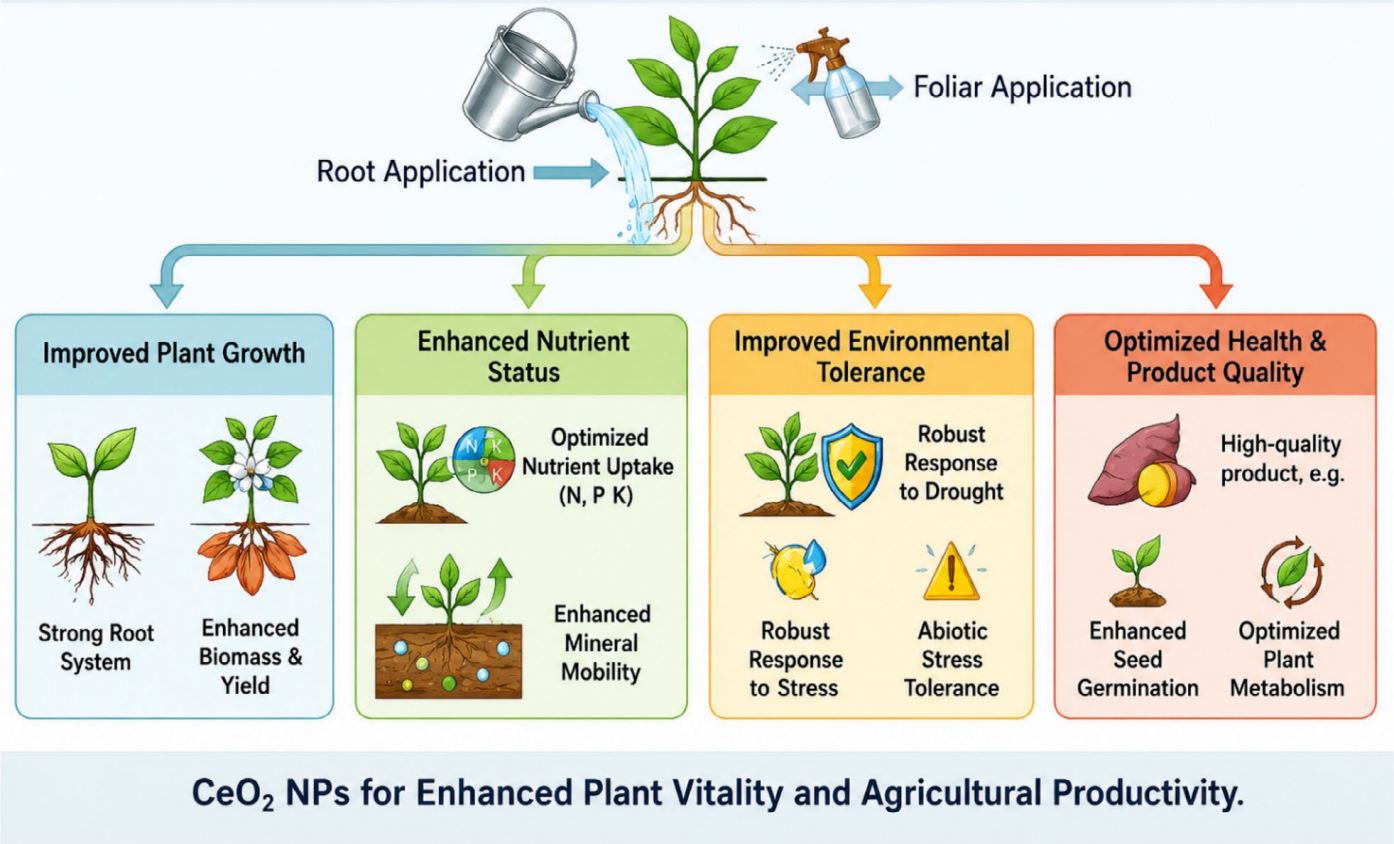
Application of CeO_2_ NPs in sustainable agriculture.

The uses of nanotechnology in agricultural systems is the enhancement of nutrient usage for pest and disease management, and it has the potential to revolutionize sustainable agricultural practices (Gupta et al. 2023). Utilizing sustainable and controlled‐release fertilizers has become increasingly important as awareness of the negative consequences of traditional fertilizers has increased (Dalei et al. 2024). Applying nanoparticles intentionally in agricultural processes may be one of the few intentional diffuse inputs of manmade nanoparticles into the environment. Along with additional advanced technologies like pest control, biotechnology (including genetics), breeding plants, disease prevention, fertilizer technology, precision agriculture, and other related fields, nanotechnology has important applications in the developing world to increase agricultural productivity (Dutta 2024). The use of nanotechnology has enormous promise for enhancing soil fertility and crop yield in sustainable agriculture. NPs can promote plant growth in harsh environments by improving nutrient uptake, regulating hormone levels, and minimizing stress‐induced damage (Hayat et al. 2023) (Figure 4). Using nano‐carriers can increase the effectiveness of supplying plants with a variety of agricultural inputs, mainly nutrients and insecticides (Irshad et al. 2024). In agriculture, nanotechnology has shown potential in addressing the problems caused by rising food demands and environmental stressors. It provides several strategies to enhance crop yields, such as applying nano‐pesticides to shield plants from diseases and nano‐fertilizers to improve soil quality, which eventually causes increased crop yields (Behl et al. 2022). To ensure sustainable agricultural production, nanomaterials must influence plant development, metabolism and yield, particularly in a range of environmental conditions (Kumar et al. 2024). Cerium is not necessary for plants but still can improve yield, seed germination, plant development, amount of chlorophyll and saccharides in plants (Ramírez‐Olvera et al. 2018). CeO_2_ NPs can also stimulate stomatal conductance or photosynthesis (Landa 2021).

Nano‐carriers can be used to enhance the efficiency of delivering a variety of agricultural products to plants, mainly nutrients and insecticides. CeO_2_ NPs have the capacity to elevate plant growth in different stresses; for example, salinity, dark chilling, heat, and excessive light (Wang, Dong, et al. 2020; Wang, Zhang, et al. 2020). CeO_2_ NPs have growth‐enhancing effects on plant growth and development and can be used to synthesize biofertilizers (Anand and Pandey 2023). Cao et al. (2017) and Zhao et al. (2015) both studied that the photosynthetic rate in soybean and corn enhanced as a result of the application of CeO_2_ NPs. CeO_2_ NPs can help crops to cope with a variety of abiotic challenges, advancing sustainable agriculture (Boora et al. 2024). Tumburu et al. (2015) found enhanced germination in *Arabidopsis thaliana* exposed to up to 500 μg/mL CeO_2_.

Nanotechnology could be used in pest management programs with effectiveness. An estimated 14% of crops worldwide are lost due to insect pests, and up to 13% are lost as a result of plant illnesses, which cost the US $2000 billion annually. Nanoparticles can be utilized to address plant diseases, can be used to deliver the pesticides in agro‐ecosystem, and plant disease forecasting; further, nano‐clays and nanofilms can be used as protective compounds against post‐harvest diseases and fruit deterioration (Dutta 2024). Under stress conditions, plants often experience significant challenges in nutrient uptake due to the decreased availability and mobility of nutrients, as well as changes in soil properties and plant physiology (Agnihotri et al. 2018). Nanoparticles help in upgrading nutrient uptake under stress conditions due to their unique properties (Nongbet et al. 2022). NPs have tiny size, large surface area, and capacity to transport and release nutrients in a regulated manner; thus, it can increase nutrient delivery to plants (Elemike et al. 2019). NPs can improve photosynthesis and protect the photosynthetic system under stress conditions (Dilnawaz et al. 2023). CeO_2_ NPs have the ability to protect the photosynthetic system from oxidative degradation. CeO_2_ NPs can reduce oxidative stress because they can act as ROS scavengers and maintain the integrity and functions of photosystems and chloroplasts (Kumar et al. 2023).

## 6 Crosstalk Among CeO_2_ NPs and Phytohormones Regulation

Hormones are chemical messengers that coordinate the cell functions of multicellular organisms (Ramlal et al. 2024). Plant hormones belong to a wider and more varied class of compounds that evolved regulatory characteristics during the evolution of embryophytes (Gasperini and Howe 2024). All of the physiological functions of plants, such as growth, defense and productivity, development, nutrition allocation, and environmental adaptation, are controlled by phytohormones. As a result, phytohormones affect all biological processes within the plant, including how it interacts with the outside world, either directly or indirectly (Fahad et al. 2015). Brassinosteroids, strigolactones, salicylic acid, jasmonic acid, and peptides are examples of phytohormones in addition to the five traditional phytohormones (auxins, cytokinins, gibberellins, abscisic acid, and ethylene), which are crucial for plant growth and stress reactions (Zhao et al. 2021). These hormones often interact with each other, a phenomenon known as hormonal crosstalk, to regulate plant stress responses. Nanoparticles can modulate this hormonal crosstalk to enhance plant tolerance to stress (Thabet and Alqudah 2024). NMs application has also been found to modulate plant developmental and defense responses by regulating endogenous levels of phytohormones such as Gibberellin, ethylene, brassinosteroids, cytokinin, auxin, nitric oxide, salicylic acid, jasmonic acid, and melatonin (Kumar et al. 2024).

Drought stress can decrease the content of macro and microelements, and the application of the content of these components in plants under drought stress changed significantly because of CeO_2_ NPs. During periods of severe drought, CeO_2_ NPs dramatically decreased chlorophyll damage. Additionally, they lessened the impacts of drought; when applied under drought stress, CeO_2_ NPs markedly increased the production of hormones such as abscisic acid and indole‐3‐acetic acid. Increased expression of the DREB1A and DREB1E genes in response to drought stress (Soleymani et al. 2025).

## 7 Efficacy of Plant‐Based CeO_2_ NPs to Alleviate Biological Stress

Living organisms like bacteria, fungi, viruses, nematodes, insects, arachnids, and weeds establish biotic stress to plants. Virus infections and chewing insects can affect the rate of photosynthesis and reduce the rate of photosynthesis per leaf area (Gull et al. 2019). Pathogens can reduce yields of crops by harming them. Application of phytosynthesized metal nanoparticles against different diseases is a unique and effective approach.

### 7.1 Antibacterial Potential of CeO_2_ NPs

**FIGURE 5:**
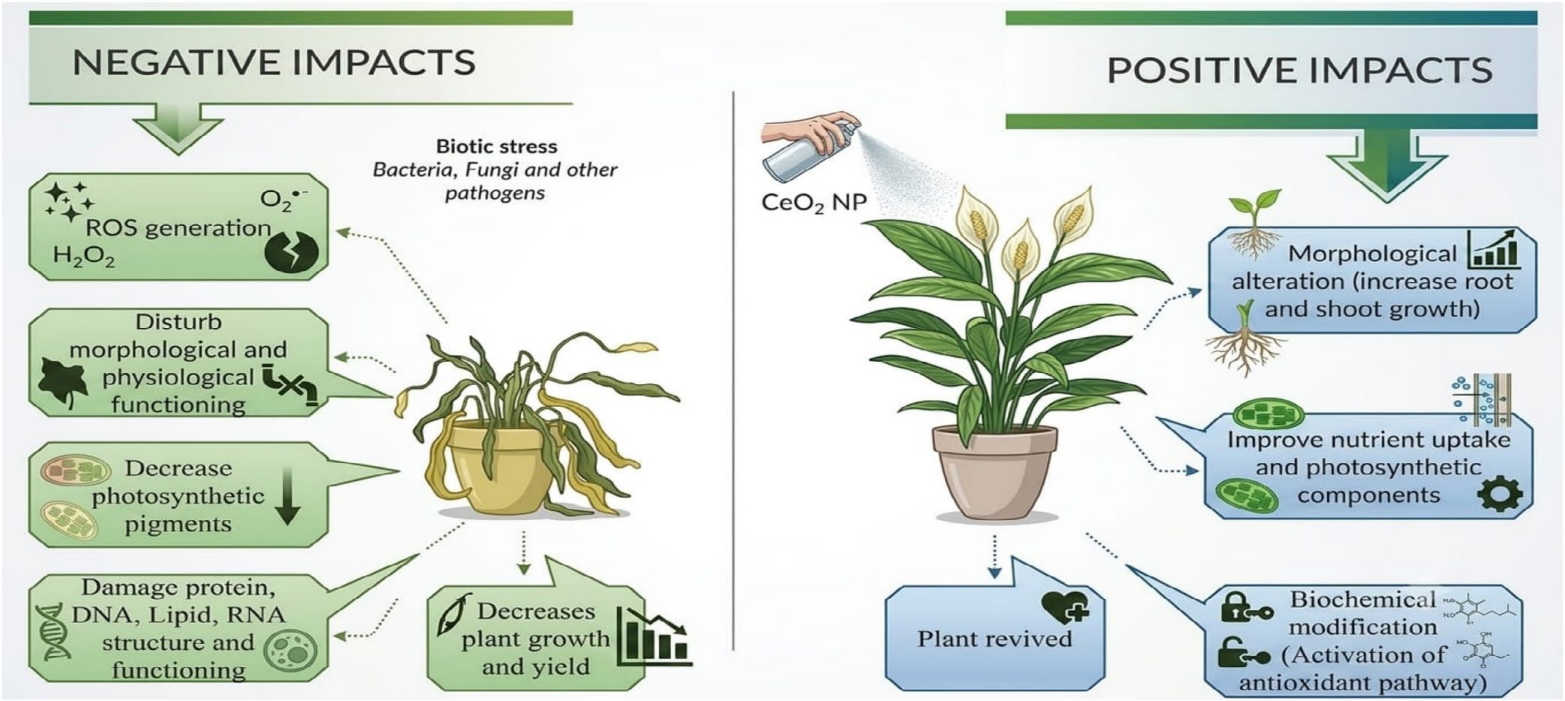
Role of biogenic CeO_2_ NPs in biotic stress in plants. In left, when a plant undergoes biotic stress, ROS are produced in excessive amounts that disturb morphological and physiological functioning, decrease photosynthetic pigments, and damage protein, DNA, lipid, RNA structures and functioning, then ultimately plant growth and yield decrease. Right, when CeO_2_ NPs are sprayed, morphological alterations, such as an increase in root and shoot growth, and biochemical modifications (activation of antioxidant pathways) occur. Nutrient uptake and photosynthetic components improve. By all these biochemical improvements, help the plant to revive. ROS (Reactive oxygen species is a by‐product of cellular metabolism. Its production leads to oxidative stress which leads to cellular damage and disease development).

Bacteria is one of the widespread microorganisms which can affect the surrounding environment in both beneficial and bad ways (Pansambal et al. 2023). In addition to alleviating symptoms, antibacterial therapy is helpful in reducing bacterial infections. The process of antibacterial activity is the regulation and reduction of the number of bacteria by knowing all features of bacterial life, including respiration, reproduction, metabolism, and nourishment. Conventional antibacterial medications, however, typically have short‐term effects and may be harmful to the environment (Pansambal et al. 2023). It has been observed that certain bacterial species are hostile to various antibiotics as a result of the prescription and overuse of antibiotics in the treatment of human and animal illnesses (Singh et al. 2020). Many researchers, however, have recently looked for a different approach to get beyond this barrier. The reason metallic nanoparticles are utilized is that they can harm bacterial cell walls by releasing reactive oxygen species (ROS), which are produced by the NPs (Singh and Bhardwaj 2024). On the other hand, antibacterial nanoparticles are thought to be minimally toxic with prolonged effects on drug‐resistant bacterial strains (Pansambal et al. 2023). Presently, CeO_2_ NPs have attracted great interest as an antimicrobial agent against bacterial pathogens (Sebastiammal et al. 2019). The precise process by which bacteria are killed is yet unclear. Nonetheless, it is hypothesized that CeO_2_ NPs primarily destroy microorganisms by causing cells to produce a large amount of reactive oxygen species (ROS) (Kumar et al. 2018). The ROS damages the membranes, disrupts the cellular compartments, breaks down the bio‐organic molecules, impairs the related processes, and eventually results in death (Nadeem et al. 2020). The general mechanism behind the antibacterial activity of CeO_2_ NPs is explained as the electrostatic interaction between the bacteria and metal oxide NPs, which damages the bacterial cell wall. Reactive oxygen species (ROS) are produced, which alter the bacterial cell wall's DNA and protein synthesis when they encounter it. Consequently, this prevents nutrients from reaching the cell, which ultimately leads to cell death (Singh and Bhardwaj 2024). Leaf extract of *C. sativa* was employed to synthesize CeO_2_ NPs using cubic fluorite‐type morphologies and a size of 52 nm. The produced NPs demonstrated potential antibacterial activity (Korkmaz et al. 2024). *Stevia rebaudiana* leaves extract produced CeO_2_ crystals having a diameter of 10–50 nm and showed antibacterial properties (Malakootian et al. 2024). In a study, CeO_2_ nanoparticles were synthesized from the leaves extract of the plant *F. religiosa*. The CeO_2_ NPs formed by this show powerful antibacterial activity (Parab et al. 2024). *Acacia concinna* fruit were used for green synthesis of CeO_2_ nanoparticles which demonstrated resilience to antibacterial properties and it has a cubic fluorite‐type shape and size of 22.7 nm (Muduli et al. 2023). CeO_2_ NPs of spherical shape and 12 nm in size were synthesized from fresh blossoms and leaves of *Matricaria recutita* , which show active potential for antibacterial activity against both gram‐positive and gram‐negative bacteria (Druzian et al. 2023). *Terminalia catappa* leaves were used to synthesize CeO_2_ NPs with a spherical shape and 98 nm in size. Reduction is caused by several phenolic and flavonoid molecules in the extract according to FTIR, and it has potential for antibacterial activity (Ray et al. 2023). *Hibiscus Rosa sinensis* flower extract was used to synthesize CeO_2_ NPs with a spherical shape examined through microscopic and spectroscopic examination. Furthermore, biologically synthesized CeO2 NPs exhibit strong antibacterial activity (Alexander et al. 2023). CeO_2_ NPs were synthesized by using the leaf extract of *Jacaranda mimosifolia* , which was characterized by XRD, EDS, and SEM techniques and have potential for antibacterial potential (Vinutha et al. 2023). Moreover, *Cassia gluca* flowers were used to synthesize biogenic CeO_2_ NPs which demonstrated antimicrobial potential against bacteria (Butt et al. 2022). CeO_2_ NPs synthesized from leaf extract of *Artabotrys hexapetalus* , having cubic morphology and size of 60 nm, characterized by SEM, FTIR, XRD, EDAX, and UV–Vis spectrometry, exhibit exceptional potential for antibacterial activity. In another research, the antimicrobial properties of CeO_2_ NPs synthesized from papaya ( *Carica papaya* ) leaf extract were examined against a number of bacterial strains using the well diffusion method, which demonstrated high potential against microbial agents, against a range of bacterial species (Joshi et al. 2023). Biogenic CeO_2_ NPs reduce stresses in plants by enhancing morphology, nutrient uptake, and biochemical modification of plants, as shown in Figure 5.

### 7.2 Antifungal Efficacy of CeO_2_ NPs

A varied kingdom, fungi are studied independently due to their distinct features that set them apart from other kingdoms, such as Animalia, Plantae, Protista, and Monera. Fungi are a vital component of our planet (Khan, Awan, et al. 2022; Khan, Li, et al. 2022; Khan, Mashwani, et al. 2022). Both microscopic and macroscopic fungi exist. A long, thread‐like structure known as a hypha makes up a fungal body, and several hyphae combine to form mycelia. There are three types of fungi: mutualistic, saprophytic, and parasitic (Wang, Dong, et al. 2020; Wang, Zhang, et al. 2020). Certain fungal species establish mutualistic connections with plants, such as mycorrhizal associations. The fungi and plant roots create a mycorrhizal association. Certain fungal species, like Glomeromycota, provide phosphorus to plants from the soil, and in return, the plants provide them with nutrition and glucose (Stürmer et al. 2018). Another example of a mutually beneficial relationship that fungal species might establish is lichens, which are a beneficial interaction between fungi and algae. Lichens are important pioneers of succession (Hawksworth and Grube 2020). Numerous fungal species cause a wide range of illnesses in people, animals, and plants, despite their beneficial effects. Rust, Smut, *Aspergillus flavus*, and Penicillium are four infamous fungal species that seriously harm crops and cause enormous losses each year (Khan, Awan, et al. 2022; Khan, Li, et al. 2022; Khan, Mashwani, et al. 2022; Sultana et al. 2024). Genus *mentha* leaf extract was used in the production of CeO_2_ NPs and has demonstrated possible antifungal qualities (Khan et al. 2024). Leaf extract from *Ocimum sanctum* L. was utilized to stabilize and reduce to prepare CeO_2_ NPs and has demonstrated likely antifungal effects (Munirathnam et al. 2023). In another study, biogenic CeO_2_ NPs synthesized from *Acorus calamus* rhizome showed notable antimicrobial potential against rust in wheat. In a different study, 10 nm spherical CeO_2_ NPs were created using fruit extract from *Punica granatum* L.; these were sufficiently tiny to pass through the nuclear membrane and access the genetic material of cells and have antifungal potential (Rajan et al. 2019). In research, *A. calamus*' rhizome extract was used to synthesize CeO_2_ NPs which demonstrated active potential to biologically and sustainably restrain the stripe rust disease, often known as yellow rust (*Puccinia striformis*), in wheat. The research observations clearly showed how CeO_2_ NPs decreased the disease index percentage of yellow rust, often known as stripe, in wheat plants. According to a paper, CeO_2_ NPs at a 30 mg/L concentration significantly enhance the physiological and morphological indices of plants (Shahbaz et al. 2022). In 2023, Alotaibi and co‐workers synthesized CeO_2_ NPs using the leaf extract of *Chenopodium quinoa* , the observations of research show strong antifungal potential of CeO_2_ NPs in wheat plants grown in the field. The results indicated that CeO_2_ NPs demonstrated considerable antifungal activity against *Ustilago tritici*. Thus, they conclude that CeO_2_ NPs could be a promising strategy to combat wheat fungal disease (Alotaibi et al. 2023). The antifungal properties of biologically synthesized CeO_2_ NPs from the leaves extract of papaya ( *C. papaya* ) were examined utilizing the well diffusion approach for *Aspergillus fumigatus* and *Aspergillus niger*. These antimicrobial agents shown remarkable efficacy against a range of fungus species (Joshi et al. 2023).

**FIGURE 6:**
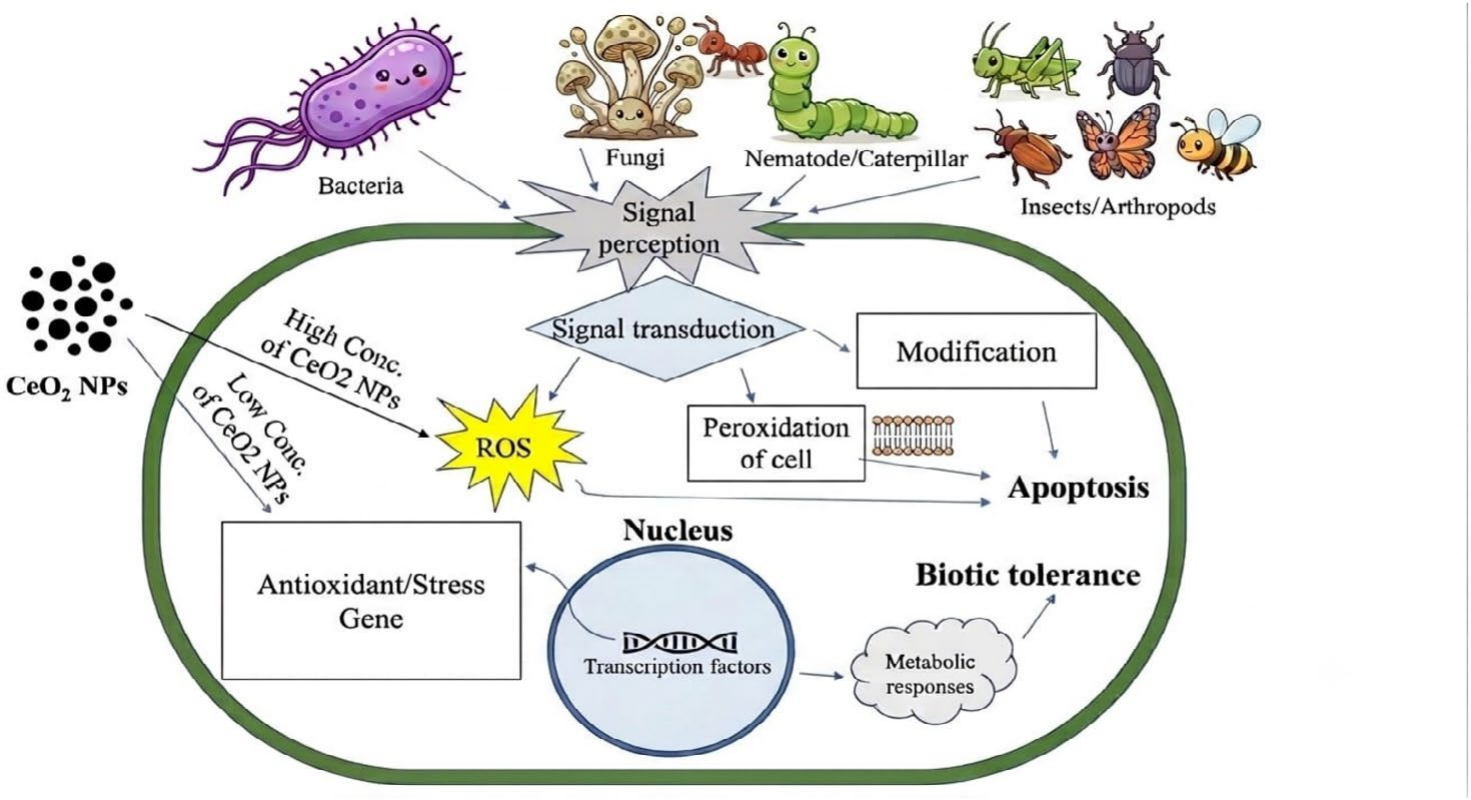
Mechanism of CeO_2_ NP mediated enhancement of plant defense responses under biotic stress. When there is biotic attack on plant, signal transduces into the cell in response to that peroxidation of cell and modification in nucleic acids occur. As a defensive response, ROS are produced in excessive amounts. These responses result in the death of the plant. When CeO_2_ NPs are applied to the plant in high concentrations, they cause toxicity, ROS are produced in excessive amounts that may lead to death but when CeO_2_ NPs are applied in low concentrations, they regulate antioxidant enzyme activity and also the stress responsive gene, then metabolic responses occur which help to develop biotic tolerance in plant.

Considering the superior antimicrobial capabilities of biosynthesized CeO_2_ NPs both in vitro and in vivo, it is not hard to think that CeO_2_ NPs mediated by plant extracts could be a great nano product to eradicate these harmful plant diseases. It is thought that biogenic NPs are more environmentally benign and biocompatible than fungicides, which harm the ecosystem. The cost of these NPs is suitable for farmers (Parveen et al. 2023). We proposed a biotic stress tolerance mechanism in Figure 6.

## 8 Role of Phytosynthesized CeO_2_ NPs in the Management of Abiotic Stress

The significant decrease in crop productivity is caused by abiotic stressors such as drought, high or low temperatures, heavy metal toxicity, and high salt conditions (He et al. 2018). Plant abiotic stress is regarded as a significant ecological limitation that eventually lowers agricultural output (Preetha et al. 2023). According to FAO statistics, abiotic stressors impact about 96.5% of agricultural land globally (Imran et al. 2021). The growth, development, yield, and seed quality of crops and other plants are adversely affected by abiotic stressors, including salt, mineral toxicity, severe temperatures (cold, frost, and heat), excessive watering (water logging), and drought (water stress) (Gull et al. 2019). The most common problem for agricultural productivity is drought stress, which significantly impairs plant development, growth, and productivity, posing challenges to the global maintenance of a sustainable agricultural system (Azeem et al. 2022). The most important limiting factor for agricultural productivity is drought stress. Drought is a periodic and persistent problem that lowers crop quality, production, and nutritional value in many places around the world. Approximately 45% of the world's agricultural land is affected by drought, and water‐deficit situations alone are predicted to generate production losses of nearly 30% by 2025 (dos Reis et al. 2016). Boora and co‐worker showed that foliar treatment of 50 ppm of CeO_2_ NPs topically to drought‐stressed wheat plants can enhance their photosynthetic indices, antioxidant enzyme activity, and relative water content (RWC). These beneficial effects can be attributable to CeO_2_ NPs' effective scavenging of ROS produced in reaction to the stress of drought, which in turn decreased the levels of hydrogen peroxide and membrane lipid peroxidation. Additionally, the expression of the stress genes increased significantly, suggesting that CeO_2_ NPs can help wheat plants develop tolerance, even at the molecular level. They conclude that using CeO_2_ NPs can improve sustainable agriculture by supporting crops in overcoming a range of abiotic challenges. Following treatment using CeO_2_ NPs under the stress of drought, all five of the drought tolerance marker genes—DREB2, ABC1, SnRK2.4, MYB33, and MYB3R—were shown to be upregulated. Among the transcription factors is the class known as MYB that assists in lessening the impacts of drought stress on crops by controlling several sophisticated biological processes, including the ROS signaling route, stomatal regulation, cell structure, and phytohormone‐mediated signaling pathway (Baldoni et al. 2015).

A crucial abiotic element that significantly impacts plant growth and productivity is salt stress, which modifies morphological, physiological, and biochemical reactions (Baloch et al. 2023; Hasanuzzaman and Fujita 2022). Excessive salinity causes osmotic stress and upsets the plants' ionic equilibrium. By altering the CO_2_/O_2_ ratio during carbon fixation, this salinity stress also impairs photosynthesis and causes plants' stomata to close. As a result, secondary stressors, including ROS production and accumulation, take place inside plant cells. Furthermore, elevated Na^+^ and Cl^−^ ions cause nutrient imbalances in salt‐stressed plants by obstructing the uptake of other vital ions such as K^+^, Mg^2+^, and Ca^2+^, which are critical for cellular metabolism (Yang and Guo 2018). In a research, Rossi et al. (2016) studied the biochemical and physiological alterations in *Brassica napus* L. (canola) cv. “Dwarf Essex” under the combined effects of CeO_2_ NPs and salt stress. CeO_2_ NPs aid in the physiology and growth of plants under salt stress; plants treated with salt had higher cerium concentrations than plants not under salt stress. CeO_2_ NP‐treated plants showed reduced stress, increased biomass, and improved photosynthetic apparatus efficiency.

The amount of heavy metals in soil that results from human activity has grown to be a worldwide problem. Chromium has an adverse impact on plant development and growth, making it extremely hazardous to plants. The most frequent effects of chromium on plants are mutagenesis, decreased yield, suppression of enzyme function, and decreased growth of leaves and roots (Singh et al. 2013). The leather industry is the biggest source of chromium contamination, followed by the textile and chromate mining industries and the electroplating sector (Tumolo et al. 2020). In a research by Ma et al. (2022), they used cerium oxide NPs, which enhanced the general development and biomass output of sunflower plants cultivated in high chromium concentrations, as well as their morphological, physiological, and biochemical characteristics. The chromium toxicity‐induced oxidative stress in plants is eventually lessened by the CeO_2_ NPs, which also increase antioxidant enzymatic activities and decrease H_2_O_2_ stress. In another research, according to the findings, CeO_2_ NPs at a dosage of 100 mg/L have a strong tolerance to withstand chromium stress and are a promising way to lower metal concentrations, boost maize growth, and advance sustainable agriculture (Latif et al. 2024).

It is demonstrated from different studies that CeO_2_ NPs are very specific in nature. It is notable that the accumulation levels, stress tolerance, and toxic effects of CeO_2_ NPs vary among different plant species. Lower concentrations of CeO_2_ NPs enhance plant growth by improving antioxidant activity, photosynthesis, and stress signaling pathways under both stress and normal conditions. However, higher concentrations of CeO_2_ NPs in plants have been found to induce phytotoxic effects. This highlights the importance of conducting research at optimal levels for different crops to benefit agriculture (Preetha et al. 2023).

**FIGURE 7:**
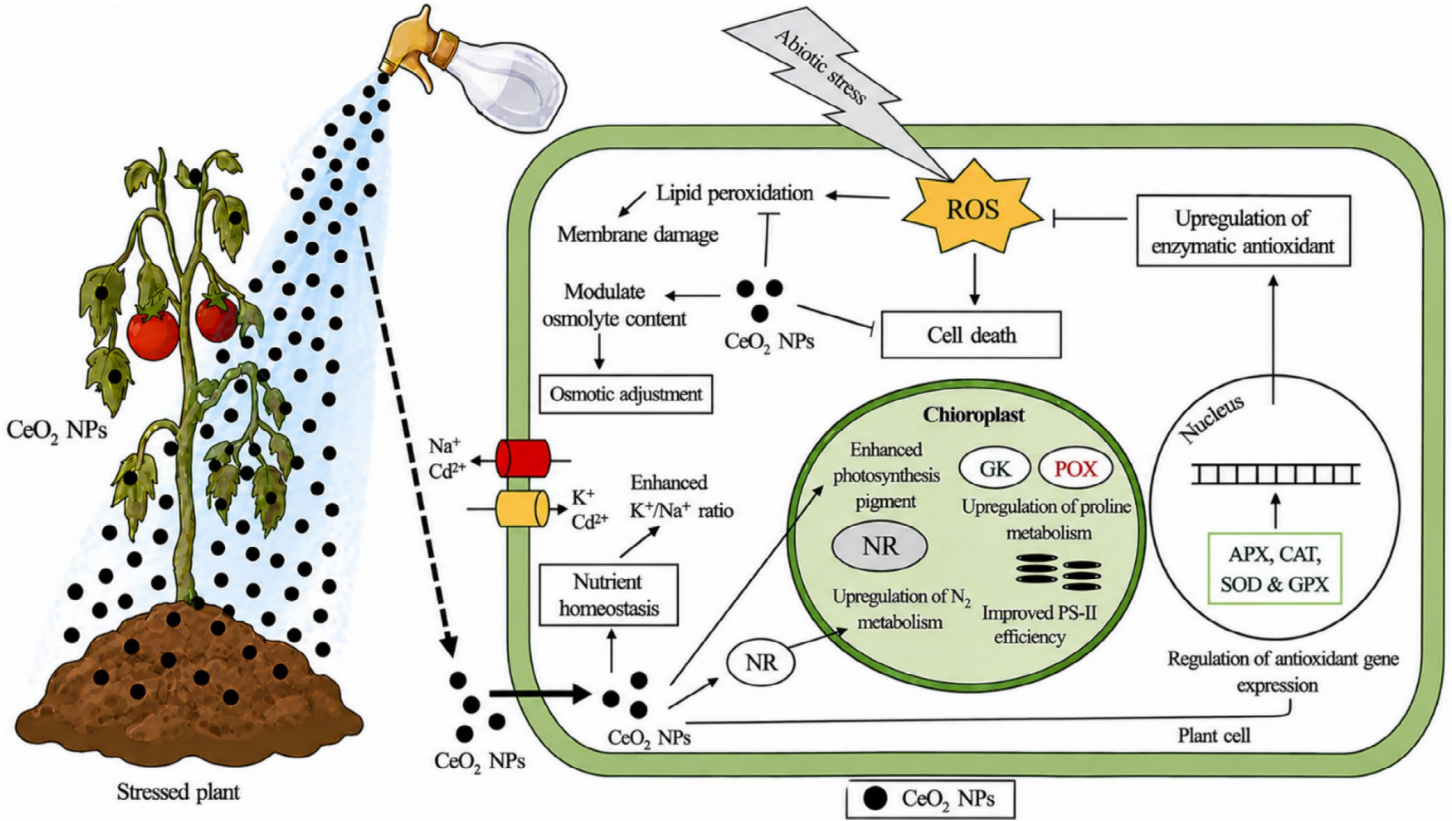
Potential application of abiotic stress tolerance in plants subjected to phytosynthesized CeO_2_ NPs. Abiotic stress sensed by plants produces ROS in large amounts, which results in the death of the plant or lipid peroxidation of the membrane. When phytosynthesized CeO_2_ NP is applied to abiotically challenged plants, it has been shown to confer resistance qualities by controlling different cellular/physiological features. The efficacy of nutrient absorption and efflux caused by membrane transporters is compromised by several abiotic stressors, but it is maintained and repaired by supplementing with green‐generated CeO_2_ NPs. CeO_2_ NPs' up‐regulation of enzymatic antioxidants helps to preserve cellular homeostasis and reduce oxidative damage brought on by abiotic stress. CeO_2_ NPs are essential for abiotic stress tolerance because they improve photosynthetic efficiency, nitrogen metabolism, and osmolyte concentrations. Red‐represented genes and proteins are down‐regulated, whereas green‐represented genes and proteins are up‐regulated. Effects evoked by NPs in a naturally occurring sequence of cellular events are indicated by black arrow lines. A line with a flat head represents the repression effect. APX, ascorbate peroxidase gene (primary antioxidant enzyme that protects cells from oxidative damage); Ca^2+^, calcium ions; CAT (anyioxidant enzymewhich defends against abiotic stress), catalase gene; GK, glutamyl kinase; GPX (glutathione peroxidase antioxidant enzymes which protects cell from oxidative damage caused by environmental stress), glutathione peroxidase gene; K^+^, potassium ions; Mn^2+^, manganese ions; Na^+^, sodium ions; POX, proline oxidase; NiR, nitrite reductase; NR, nanoparticles; ROS, reactive oxygen species; nitrate reductase; NPs, SOD (superoxide dismutase gene antioxidant enzyme which protect plant against environmental stress by breakdown of superoxide radicals, superoxide dismutase gene).

Nanotechnology emerges as a promising technology in agriculture, offering novel properties that can alleviate stress impacts and improve crop resilience through the application of nanoparticles (NPs) (Shoukat et al. 2024; Sultana et al. 2021). The potential of nanoparticles, especially metallic and metal oxide nanoparticles, to mitigate the harm caused by abiotic stress in plants has drawn attention (Ali et al. 2024) (Figure 7). By promoting physiological processes during plant growth and enhancing the quality of soil and agricultural products, nanotechnology can mitigate a variety of abiotic challenges (do Espirito Santo Pereira et al. 2021). Thus it is concluded that CeO_2_ NPs have the potential to upregulate various resistance pathways that ultimately confer plant tolerance. Mechanisms of creating tolerance and stress include these basic steps: first of all CeO_2_ NPs scavenge excessive produced ROS to maintain ROS homeostasis; next it maintain K^+^ capacity in mesophyll; after that it improves NO (nitric oxide) production; then modulation of alpha‐amylase activities take place; and lastly the lipoxygenase activity reduced to lower the oxidative damage on the membrane (Soleymani et al. 2025) and we proposed this mechanism in the following Figure 7.

## 9 Antioxidant Potential of CeO_2_ NPs

Antioxidants are compounds that can protect cells or tissues from the damaging effects of unstable molecules and free radicals that the body produces while under stress from the environment. They are sometimes referred to as scavengers of free radicals. Furthermore, Natural or artificial compounds known as antioxidants stop or slow down the harm that various oxidants, including reactive nitrogen species, nitrogen oxide species, and other unstable molecules, cause to cells (Javed et al. 2021). During biotic and abiotic stressors, antioxidants sustained oxidative stress and had tremendous potential for treating radicals caused by diverse detrimental effects (Khalil et al. 2019). Both natural and synthetic antioxidants are less effective due to their poor absorption, inability to penetrate selectively permeable membranes, and degradation, which results in low effectiveness. Furthermore, Significant antioxidant functional groups are present in plant extract‐assisted nanoparticles (NPs) with an outstanding antioxidant profile, stability, and biocompatibility (Menon et al. 2019). The defense system includes antioxidant enzymes like SOD and APX, which neutralize oxidative radicals and maintain plant's balance (Hasanuzzaman et al. 2020). Nanozymes are nanomaterials that exhibit enzyme‐like activity, mimicking the catalytic functions of natural enzyme, including oxidants and antioxidants in biological systems (Liu, Persson, et al. 2021; Liu, Xiao, et al. 2021). It was observed that CeO_2_ NPs can imitate the oxidases activities, SODs, CATs, and PODs (Lord et al. 2021). CeO_2_ NPs as nanozyme play an important role in reducing abiotic stress factors including, salinity, drought, temperature, and heavy metal stress (Preetha et al. 2023). SOD converts anion into oxygen molecules and hydrogen peroxide (H_2_O_2_) (Parab et al. 2023). Cerium oxide nanoparticle's ability to scavenge superoxide anions is resilient due to their surface oxidation state (Wang et al. 2019). POD mimetic activity in CeO_2_ NP's was determined by using a microfluidic electrochemical device with tetramethylbenzidine (TMB) and H_2_O_2_.

The chromogenic substrates TMB and H_2_O_2_ undergo redox reaction when CeO_2_ NPs are revealed by the formation of a 652 nm absorption peak and a blue color. Redox reduction was blocked when H_2_SO_4_ was added, that caused the absorbance peak to shift to 452 nm from 652 nm and the blue color to turn yellow (Alizadeh et al. 2020). CeO_2_ NPs may regulate redox reactions by changing oxidation states. They exhibit antioxidant properties that play a role in scavenging free radicals and reducing oxidative stress. Biogenic CeO_2_ NPs may be used to fortify agricultural plants from oxidative stress.

CeO_2_ NPs show enzyme‐mimetic and ROS/RNS scavenging activity mainly due to their nanoscale physicochemical properties, particularly oxygen vacancy formation and the Ce^3+^/Ce^4+^ redox cycle. At the nanoscale, CeO_2_ NPs contain mixed Ce^3+^ and Ce^4+^ states, with smaller particles having more Ce^3+^ sites and oxygen vacancies (CeO_2_ structure). This strengthens redox cycling and antioxidant activity. Results of research demonstrate that the Ce^3+^/Ce^4+^ redox couple is the key driver of biological antioxidant effects, as disrupting this cycle eliminates activity even when oxygen vacancies remain. Thus, while oxygen vacancies are structurally essential, the Ce^3+^/Ce^4+^ redox couple is the primary mechanism underlying CeO_2_ NPs antioxidant function (Nelson et al. 2016).

## 10 Plant Growth Modulation in Response to Exogenous Application of CeO_2_NPs

**FIGURE 8:**
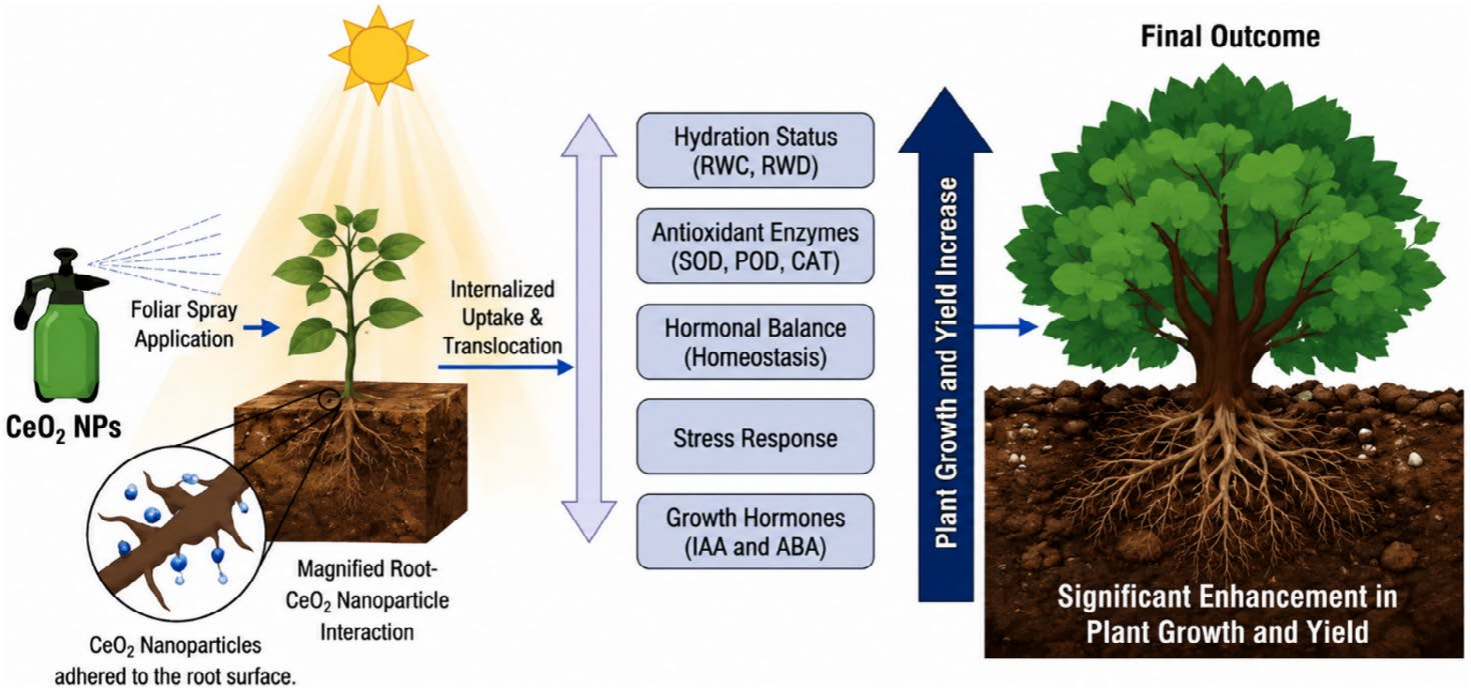
Impacts of CeO_2_ NPs on the plant. When CeO_2_ NPs are applied to a plant, they enhance the plant's internal physiological processes, such as boosting parameters such as POD (peroxidase), CAT (catalase), SOD (superoxide dismutase), RWC (relative water content), RWD (root weight density), and maintaining hormonal homeostasis such as IAA (indole‐3‐acetic acid) and ABA (abscisic acid). These biochemical improvements directly result in plant growth and yield.

Severe environmental conditions, such as pests, disease, heavy metals, salt, and drought limit plant's development and yield and have the potential to wipe out entire ecosystems. Numerous systemic signaling pathways and hormones have evolved in plants (such as ethylene, jasmonic acid and abscisic acid) to combat biotic and abiotic stressors (Wang et al. 2022). In earlier studies, scientists synthesized CeO_2_ NPs of varied sizes and forms using a variety of plants. Excessive reactive oxygen species (ROS) generation under severe stress causes biological membranes to peroxidise lipids, which ultimately causes ion leakage, reduces the efficiency of photosynthetic processes, and interferes with essential plant metabolic functions (Prajapati et al. 2022). According to new research, use of NPs may reduce the generation of ROS by activating heat shock proteins, osmolytes (such as proline, glycine, betaine, and trehalose), non‐enzymatic antioxidants (such as GSH), antioxidant enzymes (such as SOD, CAT, APX, DHAR, MDHAR, GR, GPX, and GST, etc.), and secondary metabolites produced in plants (Yuan et al. 2023). As “magic bullets,” nanoparticles can carry genes, herbicides, or nanopesticide fertilizers that target particular plant cellular organelles to discharge their contents (Siddiqui et al. 2015). Instead of using excessive amounts of chemicals to increase agricultural output, nanoparticles (NPs) can be safely applied to plants as nanopesticides and nanofertilizers (such as nano cerium) (El‐Moneim et al. 2021). Metal and metalloid nanoparticles can reduce the harmful effects caused by Abiotic stress on crops (Khan et al. 2017). Nanoparticle efficiency depends on size, shape, and concentration to interact and be absorbed by the plant system (Goswami et al. 2019). Nanoparticles can be transferred to target sites and increase their bioavailability to crops because they have small size, high surface area and strong reactivity (Banerjee et al. 2022). The reaction of nanoparticles in plants depends on plant type, age, internal and external factors (Goswami et al. 2019). Nanoparticles primarily enter through roots and leaves in to plant body. Then NPs interact with plant at the cellular and subcellular levels and cause changes in physiology and morphology of plants (Khan et al. 2019). Application of NPs can enhance seed germination. Approximately 50%–60% of seeds are lost as a result of poor technical handling. The processes from imbibition to radicle protrusion through the seed coverings are all included in germination (Carrera‐Castaño et al. 2020). Reduced germination and growth rates combined with heightened vulnerability to biotic and abiotic stressors are signs of seed degradation. Reactive oxygen species (ROS) generation and accumulation are well established to be the main causes of seed degradation in the course of ambient storage (Chandra et al. 2018; Kurek et al. 2019). It is well known that these ROS can harm macromolecules in cells. Oxidative stress, including ROS, which are extremely reactive, toxic, and capable of inducing degradative reactions of numerous biomolecules over a prolonged storage time, has been closely associated with seed aging (Adetunji et al. 2021). However, effective removal of ROS is done by production and proper functioning of a variety of antioxidants (enzymatic and non‐enzymatic) (Chandra et al. 2018). Conventional chemical and biological treatments have negative impact on the environment, potential health hazards and the emergence of disease resistance and usually don't produce long‐lasting, effective remedies, despite of all this they are widely employed (Lahlali et al. 2022). Engineered nanoparticles (NPs) have gained a lot of attention in all over the world as they have used in different fields including agriculture because of their beneficial impacts on plant development, growth and stress tolerance. A variety of metal‐based NPs can be used to revive old seeds (Kaur et al. 2021). Nanopriming is one an interesting method, in which seeds are treated with nanoparticles, it is mainly used to improve seed germination and seedling vigor under stress conditions (Shelar et al. 2024). Nanoprimers can also help to increase nutrients uptake and its utilization, and increase water intake and retention in to plant body (Rasheed et al. 2022). Pathogens like pests, fungi, viruses, and bacteria can attack the crops that pose serious risks to crop productivity and health. Nanoprimers can boost the immunity and resistance of plants against these biotic agents (Zhang et al. 2024). In a research Siddaiah et al. (2018), observed that chitosan nanoparticles showed antimicrobial action against *Sclerospora graminicola* which is the main cause of downy mildew, belonging to oomycete species. Chitosan nanoparticles can be used for the nanopriming of seeds that can exhibit increased resistance in plants with higher gene expression of alkaline peroxidase, phenylalanine ammonia lyase and polyphenol‐oxidase. Nanochitosan seed priming enhanced expression of PR1 and PR5, which are key genes in the routes of salicylic acid. According to a study, development and growth of plants is enhanced by Ce, mainly it enhanced the initial activities, growth of plants and seeds germination. Generally, 36.2% of seed germination increases by the use of CeO_2_ NPs (Ramírez‐Olvera et al. 2018). Choudhary et al. (2019) analyzed the impact of foliar application of zinc chitosan nanoparticles to maize plants and also used NPs for the seed priming. A study conducted in vitro analyzed that seed nano priming with Zn‐chitosan nanoparticle enhanced seed germination and reduces fungal development. These observations show that Zn‐chitosan nanoparticles are an effective micronutrient fortifier that help to enhance the growth of maize crops and have powerful fungicidal activity. A study conducted by Ye et al. (2020), demonstrate that growth of seedlings in saline environment is improved with application of manganese nanoparticles. Nanopriming with manganese can increase SOD (superoxide dismutase) that protect the plant from phytotoxicity and ROS damage as a result of phytotoxicity. The presence of nanoparticles in the seed may account for this behavior. In a study selenium nanoparticle as a seed priming agent demonstrated the efficacy and impact of employing on plants. The results show that nano‐priming enhanced germination, boost the development and growth of seedlings and also upgraded the nutritional value and vitality of the seedlings. Phytosynthesis of nutritional minerals is cost‐effective and eco‐friendly and can enhance the resource utilization, sustain agriculture by reducing environmental harm without immolating effectiveness (Setty et al. 2023). There are numerous effects of CeO_2_ NPs on agricultural plants. Although the effects of CeO_2_ NPs fluctuate depending on the plant species, numerous studies have shown that they can accumulate in plant roots and shoots (Anand and Pandey 2023). It has been shown in recent years that CeO_2_ NP nanofertilizer can improve plant development. Table 2 demonstrated in vivo potential application of phytosynthesized CeO_2_ NPs in the growth of plants. For example, Ahmad and Hasan (2023) produced CeO_2_ NPs by using the plant waste of *Colocasia esculenta* and applied them on mung beans. The findings indicated a substantial difference (*p* < 0.05) between the concentration of NPs and with root and shoot length of mung bean seedling growth. The length of the shoot and root was significantly increased as the concentration of CeO_2_ NPs increased. When comparing CeO_2_ NPs‐treated seeds to control (untreated) seeds, it was discovered that the treated ones had longer roots and shoots. This provided a highly notable contribution to mung bean development. Likewise, Anand and Pandey (2023) used the leaves extract of *Nyctanthes arbortristis* to synthesize CeO_2_ NPs and applied it on *Vigna mungo* . The study demonstrated the effect of CeO_2_ NPs on Blackgram ( *Vigna mungo* L.). When different groups were treated with CeO_2_ NPs like (C1, C2, and C3) at 10 mg/L., enhanced the plant's growth and development by reducing superoxide radicals (SOD) and H_2_O_2_ through enhanced antioxidant enzymes. This result show that CeO_2_ NPs have highly significant effect on the growth of *Vigna mungo* . The application of CeO_2_ NPs enhanced plant growth and development by reducing superoxide radicals and H_2_O_2_ and increasing antioxidant enzyme activity (Figure 8). In agriculture, excessive use of chemical fertilizers can be reduced by using nanoparticles (NPs), which can also enhance plant growth and seed germination in several plant species (Acharya et al. 2019). CeO_2_ NPs have shown useful effects on agricultural plants (Anand and Pandey 2023).

**TABLE 2:** In vivo potential application of phytosynthesized CeO_2_ NPs in the growth of plants.

Sr. no.	Plant	Extract	Plant model	Agronomic relevance	Mode of action	References
1	*Nyctanthes arbortristis*	Leaves	*Vigna mungo*	Phyto‐stimulatory effects, increased antioxidants	– Their forms, sizes, compositions, and atomic configurations all affect how the NPs interact with the cellular structure of plants – Increased plant growth and caused ROS accumulation	Anand and Pandey (2023)
2	*Colocasia esculenta*	Plant waste	*Vigna radiate*	Seed germination	Plant growth hormone regulation, enhancement in enzyme activity	Ahmad and Hasan (2023)
3	*Elaeagnus angustifolia*	Leaf	*Solanum lycopersicum*	Improved growth and metabolic rate	Caused metabolic alterations in addition to stimulating effects on the seedlings	Singh et al. (2019)
4	*Aloe Vera*	Leaves	*Brassica napus*	Enhance growth	Greatly raised the chlorophyll content during both the flowering and seed ripening stages	Awad et al. (2023)
5	*Petroselinum crispum*	Leaves	*T. vulgare*	Increased seedlings height	Increase the level of chlorophylls, potentially applied to agricultural plants to prevent oxidative damage	Korotkova et al. (2019)
6	*Cassia angustifolia*	Seeds	*Macrotyloma uniflorum*	Improve germination rate, root and shoot length and seed vigor	Create nanopores in seed and enhance water uptake through seed coat, modulates hormonal balance that break dormancy, trigger antioxidant signaling and accelerate starch hydrolysis which enhance radical protrusion and increase germination rate	Antony et al. (2021)

In a study, CeO_2_ NPs were phytosynthesized by using leaf extract of *Elaeagnus angustifolia*. After that, the effect of CeO_2_ NPs was investigated on the tomato seedling at different concentrations. Results showed that CeO_2_ NPs pose a positive impact on the growth of tomato (Singh et al. 2019). Another study demonstrated the impact of CeO_2_ on the development and physiology of *Brassica napus* plants. Findings concluded that foliar application of CeO_2_ NPs greatly improved morphological traits, chlorophyll and phenolic content, antioxidant enzymes, and carotenoid levels (Awad et al. 2023). A recent study documented that the phytosynthesized CeO_2_ NPs using *Petroselinum crispum* aqueous extract was applied on *T. vulgare* . Results revealed that CeO_2_ NPs, in comparison to intact materials, greatly enhanced the length of leaves and root length and also growth promoted up to 5%–11.4%. These CeO_2_ NPs were also used to protect the plant from oxidative stress (Korotkova et al. 2019). CeO_2_ NPs make changes at the biochemical level, improve the phytohormone and antioxidant system, which shows results in the form of enhancement in plant growth and improved yield. In another research, cerium oxide nanoparticles were synthesized from seed extract of *Cassia angustifolia* . Properties of CeO_2_ NPs were enhanced by selenium selenite. This phytosynthesized nanoparticles were applied on *Macrotyloma uniflorum* (horse gram) by seed priming treatment. The results prove that CeO_2_ NPs adhere and enter into the seed in nano priming and accumulate in emerging roots and shoots. Thus, it enhances the uptake of water and early growth by increasing germination and seed vigor (Antony et al. 2021).

Engineered cerium oxide nanoparticles gained attention due to an increase in their use globally. CeO_2_ NPs show both useful and harmful effects on the environment and agriculture. Proper dosage and application of CeO_2_ NPs can increase plant growth, morphology, and yield parameters. They are important for agriculture as they can eliminate harmful effects of various environmental stresses and can also improve growth parameters of plants.

## 11 Impact of CeO_2_ NPs on Soil Microbes

In the agricultural industry potential applications of CeO_2_ NPs include the management of plant diseases, enhancement of growth, mitigation of stresses, and improvement of nutrient management (Iftikhar et al. 2020). CeO_2_ NPs could be toxic to both plants and soil microorganisms if it is not properly applied. There is a complex interaction between CeO_2_ NPs, plants and soil microorganisms (Prakash et al. 2021). The interaction of CeO_2_ NPs with soil microbe is both as an antibiotics and as microbe friendly. Extensive use of CeO_2_ NPs is unavoidable release into the environment particularly the soil ecosystem during manufacturing, application and disposal. Thus these nanoparticles can modify microbial physiology, diversity, and enzymatic activities. The interaction of CeO_2_ NPs with soil microbes is difficult to understand. The nanoparticles show selective toxicity, where sensitive microbes and fungi species are suppressed or killed, whereas may survive or even spread repeatedly (Ukwattage and Zhiyong 2025). Soil microbes produce extracellular enzymes that degrade complex organic compounds in root exudates into smaller one which are more digestible molecules. CeO_2_‐NPs may hinder the activity of these enzymes by adhering to them or by generating ROS, which disable the enzymes to work properly. Study has shown that CeO_2_‐NPs decrease the cellulases and proteases enzymes activity that are important for break down carbohydrates and proteins in root exudates. When production of extracellular enzymes decreased it leads to damage soil nutrient dynamics and plant health. It can change microbial community composition and change the balance between harmful and useful microorganism in soil, for example CeO_2_ NPs shows reduction in ammonia oxidizing bacteria which play role in nitrogen cycle and results in change of nutrients dynamic in soil (Pietrzak et al. 2024). In a research CeO_2_ NPs was applied to investigate its impact on soil bacterial community in rice field. It was investigated that CeO_2_ NP inhibited plant‐growth‐promoting rhizobacteria including genera *Bacillus* and *Arthrobacter* (Pan et al. 2020) the harmful effects of CeO_2_‐NPs on soil microorganisms occur mainly by two ways oxidative stress and cell membrane disruption (Wang et al. 2017). Nanoparticles may generate oxidative stress in soil microbes through redox properties that damage microbial cellular components that includes lipids, proteins and DNA, potentially leading to cell death (Ukwattage and Zhiyong 2025). Studies have shown that CeO_2_ NPs can improve the respiration microbes as evidenced from increased metabolic quotient in the soil. In a study CeO_2−_NPs was applied at concentrations of 0, 10 and 100 mg/kg and the results indicated that CeO_2_‐NPs did not affect Carbon and Nitrogen content in the soil as well as the microbial biomass (Antisari et al. 2013). The extent to which CeO_2_‐NPs can effect on plant–microbe symbiosis depends on nanoparticle size, dosage, soil environment, and plant species.

## 12 Mechanism of Uptake, Translocation, and Accumulation of CeO_2_ NP in the Plant

Agricultural land is among the major sink to dispose nanoparticles. Plants are exposed to nanoparticles through soil or foliar application, that may cause particles to be absorbed, transfer and accumulate in plant's tissues (Prakash et al. 2020). The absorption and transport of NPs in plant are primarily attributed to foliar and root exposure. The current understanding of soil‐based exposure is lacking because of disparities in anatomical obstacles and a deficiency in technological innovations to monitor the precise route of their entry into the cells (Murali et al. 2022). Numerous factors, including nanoparticle size, physicochemical characteristics, plant physiology, and environmental stressors, influence the uptake and accumulation of nanoparticles (Rani et al. 2023). Plant uptake CeO_2_‐NPs which may lead to accumulate in different plant tissues. The accumulated CeO_2_‐NPs have deep impact on plant physiology, morphology, nutritional value, antioxidant enzymes and gene expression, and ultimately affects plant growth (Prakash et al. 2021). In a research on *Calendula officinalis* L. results indicated that foliar application of CeO_2_ NPs resulted in its higher accumulation (3200 mg L^−1^) in shoots that stimulate toxic effects and reducing shoot length. However, at lower accumulations (100 mg L^−1^) it increased shoot length and fresh weight (Jahani et al. 2019). Food safety is now a problem due to the buildup of NPs in edible crop tissues. Following root exposure, the amounts of NPs present in plant tissues typically occur in the following order: root > shoot > fruit > grains (Pullagurala et al. 2018). Because of size exclusion, nanoparticles bigger than 20 nm are unable to enter the cell wall pores. Studies on accumulation and fate of CeO_2_ NPs in plants and soil properties is critical to analyze their post effect in food chain and environment (Preetha et al. 2023). CeO_2_ nanoparticles can accumulate in plants and soil that affects both plant–microbe interactions and the food chain. As low concentrations may improve plant growth on the other hand higher doses can lead to Ce accumulation in plant tissues and edible parts potentially that cause phytotoxic effects and reduce beneficial soil microbes such as plant growth‐promoting rhizobacteria and nitrogen‐fixing bacteria. This raises apprehension about environmental impacts and possible risks to human health through consumption of contaminated edible plant parts CeO_2_ NPs readily accumulate in soil and plants tissues and can subsequently transferred through multiple trophic levels of food chain in a research trophic transfer of CeO_2_ NPs was investigated when applied on *Cucurbita pepo* L. results showed greater uptake by plants and were transferred through the food chain from soil to zucchini, then to crickets, and finally to wolf spiders. This proves that nanoparticle CeO_2_ can undergo trophic transfer and may presents a risk of food‐chain contamination (Hawthorne et al. 2014).

### 12.1 Foliar Application Method, Uptake and Translocation of NPs

Nanoparticles are taken up and move through the cuticle, the first line of defense, when exposed to foliar fluid. Water loss and solute exchange are prevented by the cuticle's waxy composition. Aqueous pores prefer the uptake of polar solutes in the size range of 0.6–4.8 nm, whereas the cutaneous route prefers the uptake of nonpolar solutes through diffusion (Prakash et al. 2020). NPs applied through foliar methods are absorbed by plants either through the stomata or the epidermis. Following uptake, both symplastic and apoplastic pathways enter the vascular tissue (Hong et al. 2021). Within plants, NPs primarily accumulate inside vacuoles and the cell wall, with vascular tissues facilitating their transport both upward and downward to other plant parts (Singh and Bhardwaj 2024). Foliar NPs enhance defenses and resistance against pests and diseases and improve the yield and quality of crops (Hong et al. 2021). Different ways are obeyed by NPs to enter in the cell. A study clearly proofs that smaller NPs that ranges from 5 to 25 nm, can pass easily through the perforated wall of cell (Hussain et al. 2019). Only the small sized nanoparticles can be absorbed due to so tiny pore channels in the cuticle and epidermis. Numerous studies have reported that when NPs are sprayed to the leaf surface, they may buildup in the xylem, phloem or epidermis (Le et al. 2022). Typically, NPs are foliar applied in agriculture where NPs accumulate and successive absorbed by the plant through stomata or cuticles. Waxy cuticle of leaf is mainly consisting of cutin, wax and pectin. It serves as the major barrier to hinder the movement of NPs into the leaf. But it also has two types of channels such as a lipophilic channel and a hydrophilic channel which play role in movement of NPs into the leaf (Pérez‐de‐Luque 2017). Both lipophilic and hydrophilic channels have diameters between 0.6 and 4.8 nm (Singh and Bhardwaj 2024). Research has shown that when CeO_2_ NPs, which range in size from 1 to 8 nm, are sprayed on the leaves of cucumber plants, they are absorbed. Foliar spray supplementation of NPs has been demonstrated to be more sustained and effective than exposure through roots or soil (Singh and Bhardwaj 2024).

### 12.2 Soil Application Method, Uptake, and Translocation of CeO_2_ NPs

CeO_2_ NPs can enter plant tissues through the root application process because they can pass through the root epidermal cell membrane. More studies have been done on NPs' effects on plant roots than on their impact on leaves. The root hair cells allow the NPs to penetrate the plant root, where they engage with the root. The initial stage of plant uptake is the CeO_2_ NPs' adsorption on the root surface (Prakash et al. 2021). Consequently, NPs move and absorb to the roots and branches of the plant (Tripathi et al. 2017). The internal structure of the plant's root consists of the vascular bundle and pericycle, whereas the cortex and epidermis comprise the external structure. At the center of the root is the vascular tissue, and the pericycle makes up the vascular column (Su et al. 2019). CeO_2_ NPs travel across the epidermis, cortex, and endodermis in the apoplastic pathway, but the casparian strips prevent them from entering the vascular cylinder, which is made up of hydrocarbons that are lipophilic (Prakash et al. 2020). Before NPs may enter the vascular tissue, they must pass via several barriers such as cuticle, epidermis, cortex, endodermis, casparian strip, and xylem (Lv et al. 2019). Nanoparticles enter through the roots or above‐ground tissue by penetrating via physiological barriers before action (Kranjc and Drobne 2019). Unluckily, there are some uncertain findings about the mechanism of absorption and transport of NPs to the root (Kurczyńska et al. 2021).

**FIGURE 9:**
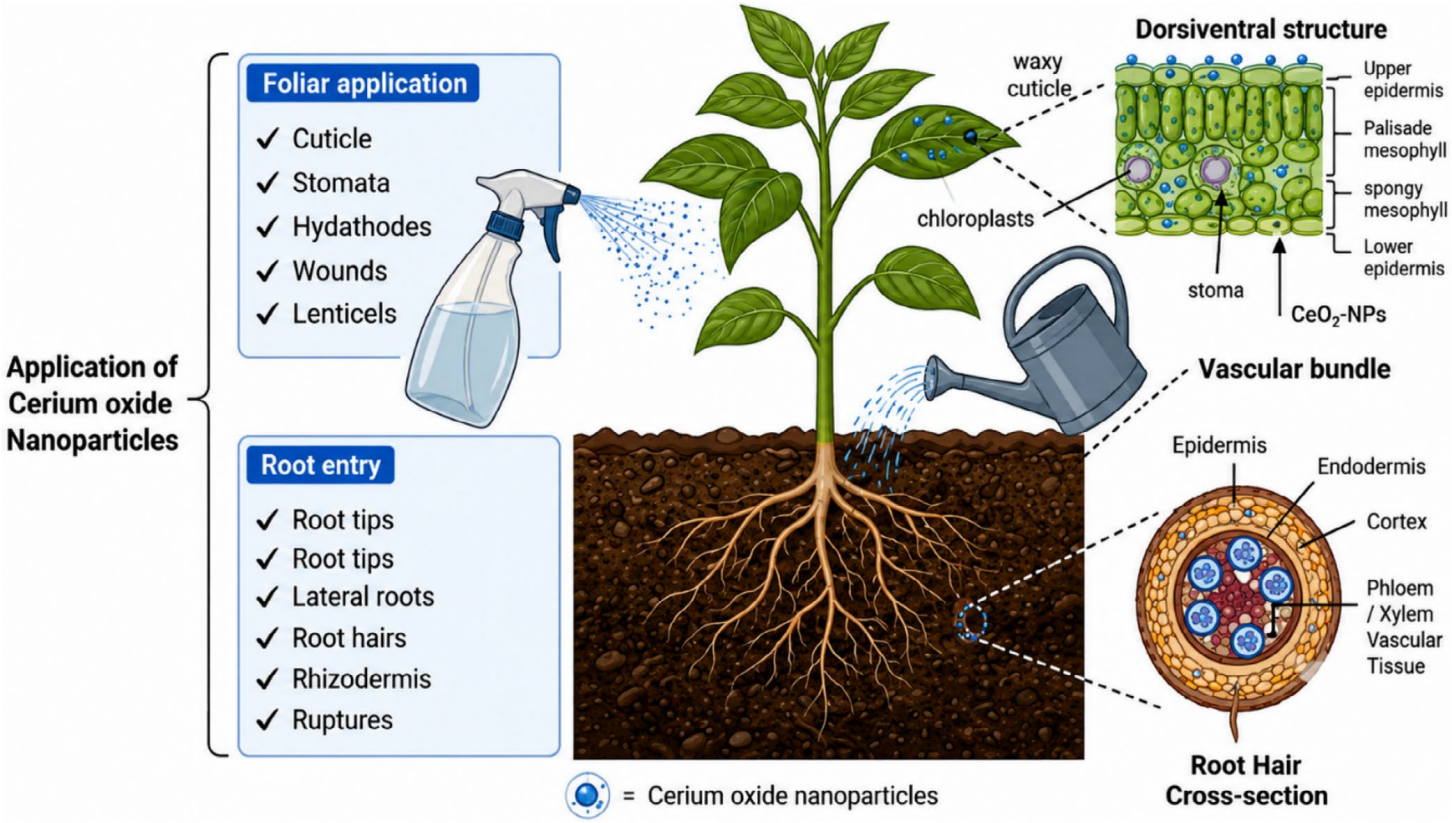
Schematic diagram showing different methods of application of CeO_2_ NPs (foliar application and irrigation application) and nanoparticles cross, transport, and translocation into the plant body.

Appealingly, the casparian strip hinders the flow of extracellular ion in plant roots through the apoplastic pathway to the xylem (Liu, Persson, et al. 2021; Liu, Xiao, et al. 2021). These strips also play a role in controlling the flow of CeO_2_ NPs and are affected by several possibilities, such as the structure of the roots, the size of the particles, their surface chemistry, and the physiological impacts on plants. According to the recent study, the concentration of NPs in plants after the root uptake is as follows: roots < seeds < fruits < shoots (Pietrzak et al. 2024). Despite the fact that the effect of CeO_2_ NPs shifts depending upon plant species, numerous studies reported that NPs build up in the roots and shoots of plants (Gui et al. 2017). Likewise, in another study, CeO_2_ NPs were synthesized by using leaf extract of *Elaeagnus angustifolia*. Afterward, the effect of CeO_2_ NPs was investigated on tomatoes and the mechanism of how NPs moved or accumulated throughout the plant (Singh et al. 2019). According to the ICP‐AES data of the study, CeO_2_ NPs were stored, absorbed, and translocated in the tissues of shoots and roots in a contrary way. Following uptake by the root cell, Ce may be transported in a unidirectional manner, along with water and minerals, to the upper tissues by xylem channels. As a result, the aggregated Ce contents in roots and shoots increased with the higher concentrations. In comparison to the plants treated with 20 mg/L of CeO_2_ NPs, the Ce accumulation in the tissues of the roots and shoots was noticeably larger at the higher concentrations of 500 and 1000 mg/L. Significant Ce buildup at lower concentrations was only observed in shoot tissues treated with CeO_2_ NPs in the current investigation. In contrast to CeO_2_ NPs, the exposure of Ce contents in the roots of CeO_2_ NPs treatments was much less than that in the lower concentrations in the shoots. When compared to treatments with lesser concentrations, the Ce content increased four to five times in the shoots treated with 500 and 1000 mg/L of CeO_2_ NPs. Higher doses of CeO_2_ were shown to increase in the shoots by two to three times. This implied that Ce would become immobile when exposed to decreasing concentrations of CeO_2_. Figure 9 shows different methods of nanoparticles, their application, and uptake in the plant body.

## 13 Molecular Mechanism of CeO_2_ Nanoparticles to Mitigate Environmental Stresses

Numerous unfavorable or even detrimental environmental conditions are continuously present for land plants. Because of the indirect or direct impact of climate change, abiotic stresses—such as salt, cold, drought, heat, heavy metals, UV radiation, ozone, and nutritional deficiencies—have a negative impact on plant development and yield (Mareri et al. 2022). According to research, metal nanoparticles significantly affect plant signaling, affecting several biological pathways and activities (Jalil and Ansari 2019). In a research by Salam et al. (2025) it was investigated that Cerium oxide nanoparticles (CeO_2_ NPs) protect maize from cobalt (Co) toxicity by increasing plant growth and photosynthesis along with reducing Co accumulation and oxidative stress. CeO_2_ NPs strengthen antioxidant enzyme activity and reduce harmful reactive oxygen species and lipid damage. Gene expression analysis indicates that CeO_2_ NPs regulate key pathways participating in metal transport, ion binding, hormone signaling, phenylpropanoid biosynthesis, and glutathione metabolism. Overall, they preserve leaf structure and enhance the plant's defense system under Co stress (Salam et al. 2025). Metal nanoparticles perform a crucial role in investigating the effects of their exposure on transcription processing in plants. The distinctive properties of CeO_2_ NPs make precise and accurate manipulation and analysis of RNA metabolism and gene expression (Van Aken 2015). Impact of CeO_2_ nanoparticles on these processes is interpreted by investigating its role in environmental monitoring, agriculture and assessing associated risks. The effect of CeO_2_ NPs in plant's genetics and molecular mechanisms have not yet been studied thoroughly. These nanoparticles may change the molecular and gene expression of plant (Francis et al. 2024).

Seed priming effect of CeO_2_ NPs on plant's response to salt stress was investigated by Khan et al. (2020), Khan, Awan, et al. (2022), Khan, Li, et al. (2022) and Khan, Mashwani, et al. (2022). After application of CeO_2_ NPs, they found that the stress linked genes that are involved in stress signaling, ion transport and antioxidant defense pathway had altered expression levels. CeO_2_ NPs being present during seed priming, results showed that certain metabolites that play a role in improving plant stress tolerance are accumulated. Furthermore, in salt stress conditions, oxidative stress was reduced by CeO_2_ NPs serving as reactive oxygen species (ROS) scavengers in plant cells. These findings showed that CeO_2_ NPs may serve as a tool for improving stress tolerance and also explain the molecular pathway how CeO_2_ NPs increase the plant resilience to salinity stress (Khan, Awan, et al. 2022; Khan, Li, et al. 2022; Khan, Mashwani, et al. 2022). NPs may change vital components such as proteins and cause biological alterations within cells and sub‐cells. In roots, NPs have been shown to alter particular proteins like protochlorophyllide oxidoreductase, 1,6‐bisphosphate aldolase, and CDK‐2. It also controls the expression of genes, including IAA‐8, RD22, and NCED3, that are important in cellular metabolism (Ghosh and Bera 2021). NPs influence the synthesis of metabolic compounds such as phytol, decane, terpenoids, organic acids and a‐tocopherol chemicals that support the plant defense system. Nanoparticle application helps in mitigating biotic stress caused by pathogens. It inhibits pathogen entry into the cell and reduces pathogen damage. It affects cell wall remodeling processes, involving enzymes like methylesterase and compounds like inositol and glycerol. NP upregulates proteins involved in protein biogenesis, such as methionine synthase, alanine aminotransferase, and serine hydroxymethyl transferase. NP triggers early defense responses, including the production of PR proteins, thiazole biosynthetic enzymes, and eukaryotic translation factors, as well as autophagy. NP influences bioenergy metabolism, affecting enzymes like formate dehydrogenase, hydroxy pyruvate reductase, and aconitase hydratase, as well as sugars and sterols. NP impacts the malate shunt, specifically malate dehydrogenase. NP inhibits stress markers like ROS (reactive oxygen species) and HSP (heat shock proteins). NP induces late defense responses, involving GST (glutathione S‐transferase) and MDHAR (monodehydroascorbate reductase) (Kumari et al. 2020).

## 14 Lethality of CeO_2_ NPs

Cerium oxide nanoparticles is widely used commercial product because it has unique physicochemical properties, and it has global annual production of approximately of 100–1000 tons/year (Dong et al. 2021). The emergence of nanotechnology presents a special chance for sustainable agriculture, but only if exposure and toxicity are sufficiently evaluated and managed (Prakash et al. 2020). CeO_2_ NPs' unique characteristics play significant role in commercial manufacturing during past 2 decades (Zhang et al. 2019). These nanoparticles have potential to promote plant growth, reducing plant stress, improving nutrient availability and also managing plant diseases (Iftikhar et al. 2020). Many of studies just focuses on the positive effectiveness of CeO_2_ NPs on plant growth and stress tolerance (Li et al. 2020). So understanding of balance between positive and negative effects is necessary for their safe application in agriculture (Ukwattage and Zhiyong 2025). Although if CeO_2_ NPs are not applied properly, it may cause toxicity to plant (Prakash et al. 2021). Research on CeO_2_ has proved that higher doses and prolonged exposure can lead to toxicity (Preetha et al. 2023). Studies on how plant seed germination is affected by CeO_2_ NPs has showed different results demonstrating plant species being play major role to the variation in findings (Prakash et al. 2021). The release of CeO_2_ NPs into and natural ecosystems is difficult to avoid. Therefore, it is very important to assess the environmental risk of CeO_2_ NPs. In a study phytotoxicity and uptake of CeO_2_‐NPs in *Arabidopsis thaliana* was studied. It was observed that CeO_2_ NPs show stimulatory effects on plant growth at low concentrations, but at higher concentrations, CeO_2_ NPs reduced growth of plant and had harmful effects on the photosystem and antioxidant systems (Yang et al. 2017). In a study, effect of CeO_2_ NPs on antioxidant potential and plant growth of *Lemna minor* was investigated. Lower concentrations such as 0.1 and 0.5 mM increased the plant growth but higher amount, that is, 1 mM caused negative impact on the growth and also signs of chlorosis showed (Zicari et al. 2018). The nutritional quality of plants can also be change by influence of absorption of minerals. In another study, barley crop quality was investigated by the application of 500 mg/kg CeO_2_ NPs. The results showed that the CeO_2_ NPs had adverse effect on the yield, quality and nutritional value of barley kernels. Amylose, sulfur and potassium content decreased while β‐glucan content remained the same (Pošćić et al. 2016). Various studies indicated that the CeO_2_ NPs have potential to alleviate wide range of environmental stresses. The boundless use of CeO_2_ NPs may cause challenges to the environment because of irregular release through industrial processes (Sendra et al. 2017). In environment, concentration of CeO_2_ was approximated to be between 0.001 and 1000 mg/kg (Roche et al. 2018). CeO_2_ NPs may preserve their nanoparticles properties such as high stability and low eco‐solubility but have harmful effects on both humans and plants (Tanino et al. 2019). They are easily transmitted to plants from the environment (Li et al. 2020), and then ultimately be transferred to humans (Dekani et al. 2019). Toxicology of NPs on plants has thus become important in recent years. For example, soybean plants could take up CeO_2_ NPs through xylem, causing negative effect on plant growth by inhibiting conversion efficiency of C5 to C3 in the Calvin cycle, and the photosynthesis is inhibited, by increasing starch granules, alter thylakoid membrane, and destroy the structure of chloroplast of soybean plants and by blocking the level of electron acceptor permanently harming the plant during photosynthesis and decreasing the production and activity of chlorophyll. CeO_2_ nanoparticles slow down the nitrification process and may even harm plant genes (Li et al. 2020). Numerous food crop's yield and nutritional value are impacted by nanoparticle toxicity. Depending on the plants and cultivars, CeO_2_ NPs have varying effects on crop quality and yield (Rico et al. 2015). For example, adding 500 ppm CeO_2_ nanoparticles to the soil enhanced the number of spikelets, spike length, and wheat yield. According to (Rico et al. 2015), adding 500 ppm of CeO_2_ NPs to the soil increased wheat grain output by 36.6%. However, the results of another study showed the opposite effect when the same NPs were applied to barley in comparable circumstances. Seedlings of barley exposed to 500 ppm CeO_2_ NPs did not produce seeds (Rico et al. 2015). CeO_2_ NPs decreased rice grains levels of sulfur (S), iron (Fe), prolamin, valeric and lauric acid, glutelin, and starch (Rico et al. 2015). In a study it was examined that on prolonged exposure to 500 mg CeO_2_/L to kidney beans plant, the root antioxidant enzyme activities were significantly reduced, at the same time it increases the root soluble protein (Majumdar et al. 2014).

Animals and vegetation may be at unknown risk from metal nanoparticles. Since the health risks associated with nanopollution are still poorly understood, it is just another man‐made environmental problem with unclear long‐term consequences. The development of treatment, monitoring, or remediation techniques is urgently needed, and authorities must impose limitations on nanopolluters (Jan et al. 2022). The toxicity of cerium particles may result in a reduction in the effectiveness of treatment in addition to the migration and accumulation of NPs inside organs, which might cause inflammatory or immunity‐related responses (Azam et al. 2025). By entering the cell, NPs can harm the plasma membranes and cell wall (Goswami et al. 2019).

## 15 Conclusion and Future Perspectives

Agriculture is necessary for humans to survive on Earth. Global agriculture is seriously threatened by biotic stress (bacteria, fungi) and abiotic stress (drought, salinity, heavy metals, heat stress), which lower food quality, crop output, and productivity. To overcome these issues, the use of chemical fertilizers increases. Chemical fertilizers are disrupting the ecosystem and negatively impacting human health. The green synthesis of nanoparticles has gained attention as a non‐toxic, environmentally friendly, and cost‐effective method. Nanoparticles of cerium oxide (CeO_2_ NPs) are used extensively, and numerous reports have documented their positive effects on the environment. This review highlights potential applications of CeO_2_ NPs to improve plant growth, photosynthetic performance, and mechanisms to reduce biotic and abiotic stress. This also demonstrates that CeO_2_ NPs influence antioxidant defense mechanisms, maintain redox homeostasis, and reduce oxidative damage. By promoting the plant's antioxidant systems to lower the build‐up of ROS in stress and trigger the production of defense genes, CeO_2_ NPs increase a plant's resistance to harmful stressors. Under both normal and stressful conditions, it has been found that lower concentrations of CeO_2_ NPs improve plant development by increasing photosynthetic parameters, antioxidant activity, and stress signaling pathways. Under natural circumstances, the application of CeO_2_ nanoparticles shows significant promise for improving plant health and raising agricultural output. However, proper concentration of CeO_2_ NPs is very important for treatment as the effect of nanoparticles on plants changes with different concentrations. Improper application of CeO_2_ NPs can affect the growth of plants, and plants can even be destroyed completely by improper concentration or application of nanoparticles.

Higher levels of CeO_2_ NPs in plants, however, have been shown to have phytotoxic consequences. Therefore, while developing an application strategy, it is crucial to consider the basic dosage of NPs, exposure length, accumulation, translocation, and mode of action on plants. Accumulation of CeO_2_ NPs in edible plant parts results in transfer to the food chain, raising important concerns about environmental safety and ecological risk. Therefore, a balanced evaluation of the useful and harmful aspects of CeO_2_ NPs is very important for implications in large‐scale agriculture.

To fully understand the mechanistic potential of plant mediated CeO_2_ NPs, more research is needed to improve the purity and yield of nanoparticles and to evaluate the toxicity and environmental impact of CeO_2_ NPs. Furthermore, as there is little research on the subject, thorough studies are required to evaluate the significance of phytosynthesized CeO_2_ NPs in abiotic stress regulation in agriculture. On the basis of Table 1 outcome we recommend having high antimicrobial potential. In future it can be helpful in alleviation of abiotic and biotic stresses in plants. There is a need for comprehensive studies to assess the implications of CeO_2_ NPs on the plant and their potential effects on the food chain. Ce, have the potential for genotoxic effects in both plants and animals; future research must develop into these aspects to ensure the safety of CeO_2_ NPs, particularly when they are consumed through edible plant parts.

## Funding

The authors have nothing to report.

## Disclosure

The authors affirm that no generative AI tools were used in the writing, analysis, or editing of this manuscript. All content was produced through the authors' original scholarly efforts.

## Ethics Statement

The authors have nothing to report.

## Conflicts of Interest

The authors declare no conflicts of interest.

## Data Availability

The authors have nothing to report.
